# Revealing the component structure of the world air transportation network

**DOI:** 10.1007/s41109-021-00430-2

**Published:** 2021-11-24

**Authors:** Issa Moussa Diop, Chantal Cherifi, Cherif Diallo, Hocine Cherifi

**Affiliations:** 1 Section of Computer Science, University Gaston Berger, Saint-Louis, Senegal; 2 DISP Lab, University of Lyon 2, Lyon, France; 3 Departement of Computer Science, University of Burgundy, Dijon, France

**Keywords:** World air transportation network, Community structure, Component structure, Mesoscopic structure, Core structure

## Abstract

Air transportation plays an essential role in the global economy. Therefore, there is a great deal of work to understand better the complex network formed by the links between the origins and destinations of flights. Some investigations show that the world air transportation network exhibits a community and a core-periphery structure. Although precious, these representations do not distinguish the inter-regional (global) web of connections from the regional (local) one. Therefore, we propose a new mesoscopic model called the component structure that decomposes the network into local and global components. Local components are the dense areas of the network, and global components are the nodes and links bridging the local components. As a case study, we consider the unweighted and undirected world air transportation network. Experiments show that it contains seven large local components and multiple small ones spatially well-defined. Moreover, it has a main global component covering the world. We perform an extensive comparative analysis of the structure of the components. Results demonstrate the non-homogeneous nature of the world air transportation network. The local components structure highlights regional differences, and the global component organization captures the efficiency of inter-regional travel. Centrality analysis of the components allows distinguishing airports centered on regional destinations from those focused on inter-regional exchanges. Core analysis is more accurate in the components than in the whole network where Europe dominates, blurring the rest of the world. Besides the world air transportation network, this paper demonstrates the potential of the component decomposition for modeling and analyzing the mesoscale structure of networks.

## Introduction

Air transport plays an essential role in the current context of globalization by reducing the distance between countries. Whether it is for the movement of millions of people or goods, thousands of flights are made per day, impacting the global economy and even public health (Colizza et al. 2006). Subsequently, the spread of the COVID-19 pandemic is mainly due to the world air transportation network. This pandemic leads to the bankruptcy of several airlines and affects the tourism and trade industry. That is why researchers have been interested in the air transportation network for a long time to study its structure, dynamics, and robustness (Zanin and Lillo 2013).

One can consider three levels of analysis of a network (macroscopic, microscopic, mesoscopic). Macroscopic analysis characterizes the entire network topology through a set of global measures. Microscopic studies investigate the network properties at the node or link level. Mesoscopic analysis concerns groups of nodes or links sharing similar features. There are two popular mesoscopic structure models: the community structure and the core-periphery structure. Although there is no consensus on their definition, both are related to the non-homogeneous density observed in real-world networks. The common understanding is that communities are dense areas of the network sparsely connected (Fortunato and Hric 2016). The core-periphery structure considers that a network comprises a dense, cohesive core and a sparse, unconnected periphery (Borgatti and Everett 2000). These mesoscopic structures are observed in most real-world networks. While they find numerous applications and explain a broad range of phenomena in networked systems, none of them is well-suited to disentangle the local interactions of nodes within their dense area from their global interactions with the other dense areas of the network. Inspired by the works reported in Ghalmane et al. (2019), Guimera et al. (2005), we propose a new mesoscopic representation of a network, called the component structure. Indeed, the authors show that considering the interplay between intra-community links and inter-community links in a modular network allows defining effective centrality measures. While these works focus on computing centrality measures in modular networks, our proposition is more general. Our goal is to distinguish between the local and global influence of various groups of nodes or links. To this aim, the proposed model decomposes a network into local components and global components. The local components are isolated, dense parts of the network that can be uncovered using a community detection or a multicore detection technique. The global components are the subnetworks joining the local components. One extracts them easily, based on the links between local components. Although it also relies on dense areas of the network as the community or core-periphery structures, the component structure offers a complementary view of the network mesoscopic organization. Indeed, components are isolated networks that can be analyzed separately. Furthermore, as the overlapping community structure, it does not operate a partition of the network. Indeed, in overlapping communities, a node can belong to multiple communities. In the component structure, a node can belong to a local and a global component. It is the case of the nodes linked to the other groups.

Our work departs from recent studies focusing on robustness (Lordan and Sallan 2019), and multilayer modeling (Lordan and Sallan 2017; Dai et al. 2018) of the air transportation network. Our main concern is to use the world air transportation network as a case study to evaluate the ability of the component structure representation to get a better understanding of the network mesoscopic organization. Therefore, we concentrate on the monolayer network representation of the world air transportation network. However, one may note that the component structure can be interpreted as a multilayer representation where the layers are the local components. Nodes and links connecting the various local components form the global components. The main contributions of this paper are as follows:

1. We introduce an alternative mesoscopic network structure called the component structure where local components are dense subnetworks, and the global components account for their interactions.
2. We use the component structure to study the structure of the world air transportation network. The local components characterize its regional organization, and the subnetworks forming the global components represent their interactions.
3. The regional characterization of the world air transportation network is not based on geographical considerations, but it relies on the density of the interactions.
4. This representation allows us to distinguish the regional impact of an airport from its influence worldwide.
5. We perform an extensive topological analysis of the component highlighting the regional and inter-regional differences of the world air transportation network.

The rest of the paper is organized as follows. Section "Litterature review" reports a review of related studies of the air transportation network. Section "Component structure of a network" introduces the definition of the component structure, and it gives an algorithm to uncover it. Section "Uncovering the dense parts of the world air transportation network" describes the data used in the experiments and examines the network community structure. Section "Local component structure of the world air transportation network" reports the analysis of the local component structure and section "Global component structure of the world air transportation network" analyzes the global component structure. Comparisons with the whole world air transportation network are reported in section "Comparison of the world air transportation network with the large components". Section "Degree centrality analysis" presents the results of a comparative analysis of the degree centrality of the components and the world air transportation network. Section "Core analysis" discusses the results of the core structure analysis. Finally, we conclude in section "Conclusion".

## Litterature review

One can distinguish three levels of study of the air transportation network: worldwide, regional and national. Based on this classification we present some influential contributions. For more information the reader can refer to the following surveys (Rocha 2017; Lordan et al. 2014).

Several studies have been devoted to the properties of *the worldwide air transportation network*. In Guimera and Amaral (2004) Guimera et al. conduct the first exhaustive analysis of the world air transportation network. Considering airports as nodes and direct connections as links, the authors show that the degree and betweenness centrality exhibit a power-law distribution. Their work also reveals that highly connected cities are not the ones with high betweenness centrality values. This feature contradicts the classical preferential attachment formation model with a geographical distance constraint (Yook et al. 2002). They propose a new model which generates nodes with large betweenness and small degree based on geopolitical considerations. Indeed, only a few airports in each country are permitted to connect to airports of other countries, regardless of the geographical distance.

In Guimera et al. (2005), the community structure is investigated. Results show that communities correspond to geographical areas. Seven city roles are defined based on the proportion of intra-community links and extra-community links. Three roles distinguish the highly connected nodes. “Provincial hubs connect cities in their community. The “Connector hubs” are in the majority linked to cities outside their community. Finally, “Kinless hubs” share their links uniformly with all the communities. In the four non-hub categories, the “Ultraperipherical” nodes have no inter-community links. “Peripherical” nodes share most of their links with nodes in their community. “Nonhub connector” nodes have many links with nodes outside their community, and “Nonhub kinless” nodes share their links uniformly with all the communities. The main contribution of these works is to highlight the importance of geopolitical considerations in the formation mechanism of the air transportation networks and the various role of cities.

Inspired by fractality analysis, in Sun et al. (2017) the authors consider six types of nodes from fine-grained to coarse-grained granularity: airport, city, spatial areas of 100 km diameter around hubs, spatial areas of 200 km diameter around hubs, sub-national territory, country. Links are direct connections between nodes. The network analysis shows that all networks are small-world and disassortative. Furthermore, the clustering coefficient increases and the average path length decreases as the aggregation level varies from fine to coarse. The community structures uncovered by Louvain are quite consistent. It contains about ten communities corresponding to different geographical boundaries.

Cheung et al. (2020) explores the evolution of the world air transportation network during the period 2006–2016. In this weighted network, nodes are airports, and the total number of passengers per year weights the direct fly links. The authors propose a new metric called Global Airport Connectivity Index, measuring the importance of airports in global passenger movements. It combines degree, closeness, eigenvector centrality measures, flow betweenness, and an indicator of regional importance. Building on the work of Guimera et al. (2005), they classify the airports into regional hubs or global hubs, depending on their embeddedness in their community and their Global Airport Connectivity Index. Results show that the average degree and the density increase over time. Furthermore, North America, Russia, and China focus on developing regional hubs, while West Europe and the Middle East concentrating on emerging global hubs.

Some studies focus on *the regional air transportation network*. In Lordan and Sallan (2017), the authors investigate the European airport network where nodes are European cities, and links account for direct flights. It appears that the degree follows a two-regime power-law distribution and that the network possesses the small-world property. Using the k-core, they decompose the network into three layers: the core (max k-core), the periphery (k-core of degree one), and the bridges between the core and the periphery. The analysis shows that the core contains the global European cities. The leisure air travel origins and destinations compose the bridges. Finally, the local destinations constitute the periphery. A robustness study shows that the network is more vulnerable to the isolation of a combination of core and bridge nodes.

In Dai et al. (2018), the authors investigate the evolution of Southeast Asian air transportation over the period 1979–2019. Results indicate that the number of hubs increases in this scale-free network. Disassortative behavior increases with time due to a more pronounced hub-and-spoke configuration of small airports for better accessibility. Decomposition of the network into a core-bridge-periphery structure shows that the core comprises the regional capital cities, the most economically vibrant secondary cities, and tourist destinations. The periphery cities are in remote areas with declining connectivity. High volatility over time characterizes the bridge nodes. The number of connections and passengers increases mainly in the core layer and the bridge layer at the end of the 20th century.

Lordan and Sallan (2019) use the Official Aviation Guide (OAG) subdivision to partition the world air transportation network into seven global regions (Africa, Asia, Europe, Latin America, Middle East, North America, Southwest Pacific). In these networks nodes are cities and links represent direct connections. Analysis shows that these small-world networks exhibit a two-regime power-law degree distribution. Targeted attack experiments based on the network’s decomposition into core, bridge, and peripheral nodes show that regional networks with a large core are more resilient than networks with a smaller core.

Much more works concern *the national air transportation network* of major countries (US, China, India, Brazil) Wandelt et al. (2019). We summarize the main findings of the following related studies where nodes are airports and links represent direct flights. The common characteristics of all these networks is that hey all share the small-world property and are disassortative. In Guida and Maria (2007) the authors investigate the Italian network during three non-overlapping periods (June 1, 2005, to May 31, 2006–July 16 to August 14, 2005–November 2005). Results are consistent across networks. Indeed, the degree distribution and the betweenness centrality follow a double Pareto law. Their findings also suggest that the networks exhibit a fractal structure. Furthermore, the clustering coefficients are comparable and lower than those observed in a corresponding random network. It also appears that some highly connected airports have a small betweenness centrality.

In Bagler (2008) the authors investigate the network of India. They consider an unweighted directed network and a network weighted by the number of flights by week. The unweighted network has a truncated power law degree distribution. It is disassortative with a clustering coefficient one order of magnitude higher than the corresponding random network. The weighted network presents a hierarchical structure. Its analysis shows that highly connected airports share almost all the traffic, forming high traffic corridors.

The Chinese network has been extensively studied (Du et al. 2017, 2017; Yang et al. 2021). In Wang et al. (2011) the authors show that its structure diverges from other national networks. Indeed, the exponential is a better fit than the power-law for the degree distribution. The explanation lies in the influence of the three main metropolises (Beijing, Shanghai, and Guangzhou). The network is disassortative with highly connected cities surrounded by poorly connected cities with direct links. This phenomenon gets more pronounced as the degree increases. Indeed, small airports in China tend to supply direct links to the top hubs bypassing the less developed regional ones.

Extensive research on the topology and the dynamics of the U.S. air transportation network have been performed (Jia et al. 2014; Xu and Harriss 2008). In Cheung and Gunes (2012) the authors analyze its evolution over the period 1991–2011, and the study reported in Siozos-Rousoulis et al. (2021) concerns the period 2001–2016. Overall, one does not observe considerable changes in the topological properties of the networks. The number of airports and flight routes has increased according to user demand. The network exhibits a truncated power law degree distribution. It is highly disassortative, and this trend grows with time, suggesting a pronounced evolution towards a hub and spoke structure over time. Similarly, with the world air transportation network, some high betweenness centrality nodes such as Anchorage cannot be considered hubs. The clustering coefficient decreases over time, and the average shortest path increases. It is in line with hub and spoke organization where peripherical airports connect to hubs providing long-distance flights.

Several papers are devoted to the analysis of the Brazilian air transportation network (da Rocha 2009; Costa et al. 2018; Oliveira et al. 2020). In Couto et al. (2015), the authors consider three networks: the network of national flight, the network of international flight and the network with both type of flights. The network is scale-free. Six communities corresponding to geographical areas (“North”, “Center/North”, “Northeast”, “Minas Gerals”, “Southeast”, “South/West”) are discovered by Louvain. The network is not resilient to targeted attack. Viracopos and Guarulhos are the key airports in the national network and for international connections, and they have the largest values of degree centrality and betweenness centrality. In addition, the number of routes decreases while the number of passengers increases, causing a higher level of occupation of aircrafts.

The analysis of the Australian network (Hossain and Alam 2017) shows that it is scale-free. Its clustering coefficient is higher than its random network version indicating a cohesive network where passengers can be easily rerouted. The average path length suggests that, on average, a passenger can reach every destination in 3 flights. Most of the traffic goes through an interconnected group of high-degree nodes surrounded by low-degree neighbors. Centrality analysis shows that the more connected nodes do not necessarily exhibit the largest betweenness and closeness centrality values.

This literature review’s main findings are that the topological properties are pretty consistent across the various levels of studies. Indeed, the degree and betweenness centrality exhibit a heavy tail distribution. Networks are small world and disassortative, with most nodes poorly connected to a few highly connected nodes. Nevertheless, the preferential attachment mechanism is not sufficient to explain the formation of the networks. One needs to consider geographical constraints (borders) and political and economic issues to get a better understanding of the network’s topology. The clustering coefficient is higher than in random networks, and the average path length allows to join any destination in few hops. Another critical finding concerns the mesoscopic properties of the world air transportation network. Some studies demonstrate that it exhibits a community and a core-periphery structure.

## Component structure of a network

This section introduces the definition of the component structure and an algorithm to uncover it based on the community structure.

### Definition

Community structure and core-periphery structure models assume that the density is not homogeneous in a network. Dense areas form either the communities or the core elements of the networks. These two mesoscopic representations share a different view of the remaining nodes or links. In the community structure approach, communities are supposed to be sparsely connected by inter-community links. In the core-periphery structure, peripherical nodes are poorly connected to each other and with core nodes. To define the component structure, we retain both approaches’ common points of view, i.e., the network contains dense areas. Those dense areas are localized in the network. Indeed, the vast majority of nodes interact with nodes contained in their community or core. That is the reason why we call them local components. Indeed, they share information with the rest of the network through a set of proxy links and nodes that have a more global view of their environment. These subnetworks tie together the local components. Consequently, the definition of the component structure is quite simple. A network contains two sets of subnetworks: 1) The dense parts of the network form the local components 2) Nodes and links shared by any two local components form the global components. Note that once the dense areas are extracted, global components identification is straightforward. Furthermore, one can exploit the various definition of dense areas proposed either in the community detection literature or the multi-core-periphery studies to extract the local components. In the following, we propose an algorithm that uses the community structure approach.

### Component structure detection algorithm

Building on the work of Ghalmane et al. (2019), we propose a component structure extraction algorithm exploiting the community structure. Remember that a network is decomposed into local components and global components. The local components are isolated dense parts of the network. The global components are subnetworks joining the local components.

The algorithm to uncover the component structure of a network proceeds as follows:

1. Uncover the dense part of the network: Use a community detection algorithm to uncover the community structure.
2. Extract the local components: Remove the inter-community links from the community structure to form the local components.
3. Extract the global components: Remove the intra-community links from the community structure, and the subsequent isolated nodes.

Note that this representation is redundant. Indeed, a node can belong simultaneously to a local component and to a global component. Such nodes at the frontier of the communities are important locally and globally.

**Fig. 1:**
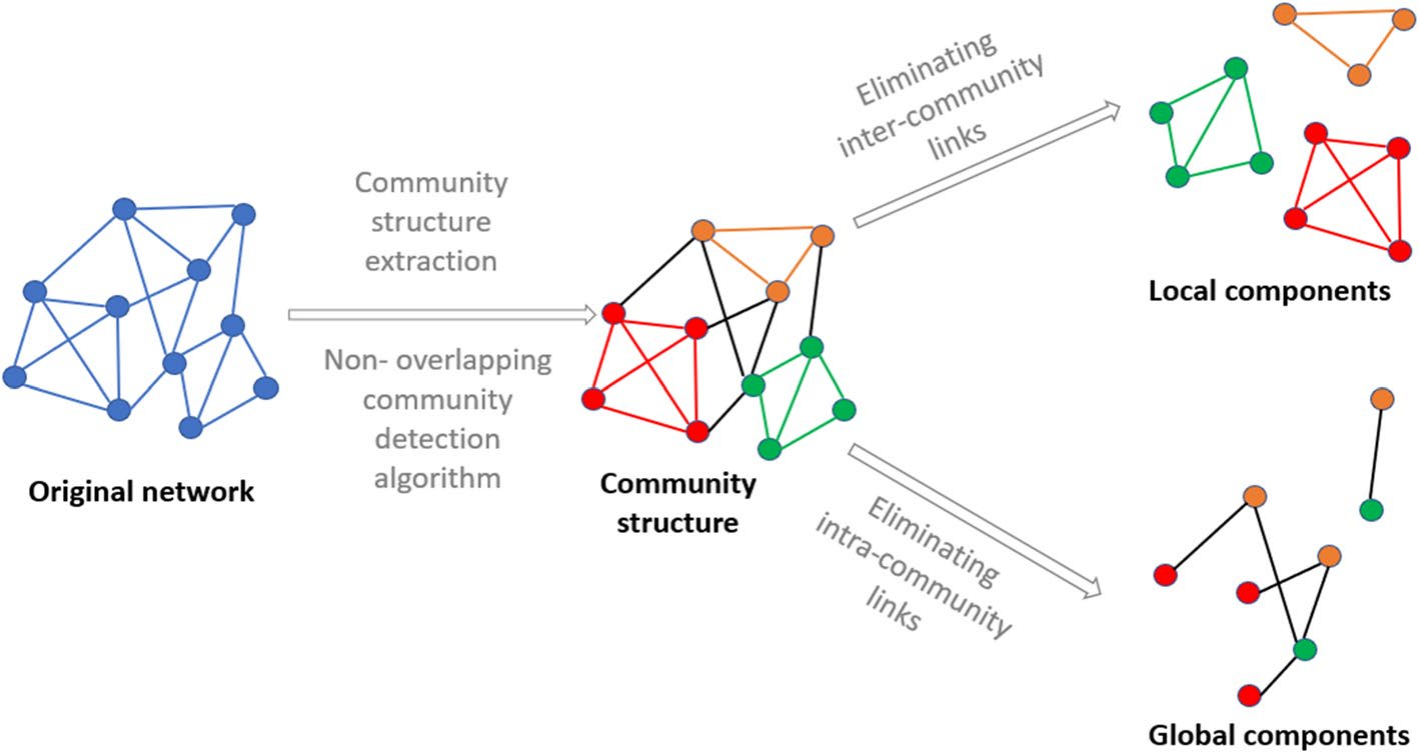
The component structure detection algorithm. First, the community structure of the network is uncovered. Second, the local components are obtained by eliminating inter-community links from the community structure.Third, the global components are obtained by eliminating intra-community links and subsequent isolated nodes from the community structure

Figure 1 illustrates the decomposition process of a network into its components on a toy example. First, one uses a community detection algorithm to partition the network into a set of non-overlapping communities. Inter-community links joining nodes in different communities are black. Nodes and intra-community links that bind nodes in the same community share the same color. We observe three communities respectively colored in red, yellow, and green. Removing the inter-community links allows us to isolate the three local components. Each community forms a local component, and it carries only local information. One obtains the global components removing the colored links from the community structure (intra-community links) and the subsequently isolated nodes. Global components act as bridges between the dense areas of the network. They are important actors in the information diffusion between the various dense parts of the network. As components are isolated sub-networks of the initial network, one can proceed to any type of topological analysis.

## Uncovering the dense parts of the world air transportation network

In this section, we present the data set used in the experiments. We perform a comparative analysis of the community structure uncovered by two popular community detection algorithms used to extract the dense parts of the network.

### Data

Information on world flights has been collected from FlightAware. The data covers six days (between May 17, 2018, and May 22, 2018), ensuring the inclusion of less frequent connections (Alves et al. 2020). Nodes represent airports, and links represent direct flights between two airports. For the sake of simplicity, the network is undirected and unweighted. However, one can consider weighted and directed networks using the appropriate analysis tools. The network contains 2734 nodes and 16,665 links. Table 1 reports its basic topological properties.

**Table 1:** Basic topological properties of the world air transportation network

***N***	|***E***|	***d***	***L***	***μ***	***ζ***	***λ***	**η**
2734	16665	12	3.86	0.004	0.26	−0.05	0.09

*N* is the network size. |*E*| is the number of edges. *d* is the diameter. *L* is the average shortest path length. $\mu$ is the density. $\zeta$ is the transitivity also called global clustering coefficient. $\lambda$ is the assortativity also called degree correlation coefficient. $\eta$ is the hub dominance

### Community detection

The first step of the component structure detection algorithm consists in uncovering the dense part of the network using a community detection algorithm. Community detection is a very active field of research. Classically, the goal is to build a network partition into well-separated groups of nodes densely connected. In some situations, especially in social networks applications, nodes can belong to several groups. Therefore, overlapping groups are also investigated. Numerous algorithms inspired by different paradigms have been proposed so far. They are based on different methods such as modularity optimization, random walk or label propagation, among others. For complete coverage on this fundamental issue, one may refer to surveys reporting comparative studies, challenges, and open problems (Orman et al. 2012; Fortunato and Hric 2016; Javed et al. 2018; Papadopoulos et al. 2012; Chunaev 2020; Cherifi 2018; Cherifi et al. 2019).

In our experiments, we use two influential non-overlapping community detection algorithms, Louvain (Blondel et al. 2008) and Label propagation (LPA) (Raghavan et al. 2007). The goal is to compare the uncovered community structures. Indeed, the community structure can be represented by various partitions with competitive quality score (Kirkley and Newman 2021).

Louvain is a modularity-based algorithm. Its fundamental principle is to maximize the modularity at each call of the procedure. Two steps are repeated iteratively. Initially, each node represents a community. Then, one computes the gain in modularity obtained by moving each node in the community of its neighbors. The process stops when no modularity improvement is possible. The second step consists of creating a new network from the communities identified in the first step.

LPA is based on the label propagation method. It works as follows. At the initialization, assign a label at random to each node. Labels change at each iteration. Indeed, the nodes adopt the most common label of their neighbors until label change is no more possible. Groups of nodes sharing the same label form the communities uncovered by LPA.

### Community structure analysis

We compare the community structures using two quality score functions: modularity and mixing parameter (Jebabli et al. 2018). Modularity compares the proportion of intra-community links with the ratio of expected links in a random network model. Typically, one considers that the community structure with the highest modularity corresponds to the best partition. The mixing parameter is the fraction of inter-community links. It reflects a strong community when it is near zero. The community structure with the smallest mixing parameter is considered the best one. Table 2 reports the results related to the quality metrics of the community structures uncovered by the two algorithms. The modularity values show that both community structures are well-defined. Moreover, it reveals that the community structure uncovered by Louvain is of better quality. The mixing parameter values indicate a low proportion of inter-community links. It corroborates the strong community structure measured by modularity.

Although both algorithms uncover well-separated and dense communities with few inter-community links, the community structures differ. Indeed, Louvain reveals 27 communities, while the community structure identified by LPA contains 130 communities. This result is not unusual. A wide range of different community structures can lead to community divisions with comparable quality scores. Even though these partitions differ, they are consistent. They relate through a set of “building blocks” that usually appear together in the same community (Riolo and Newman 2020). The Normalized Mutual Information (NMI) measures the information shared by two partitions. It is widely used to compare community structures (Orman et al. 2012). When the NMI is equal to one, the two partitions are identical. When it is null, the two partitions are independent. Its high value reported in Table 2 confirms that both community structures have a lot in common.

**Table 2:** Quality metrics of the community structures uncovered by Louvain and LPA community detection algorithms: modularity, mixing parameter, NMI

	Modularity	Mixing parameter	NMI
Louvain	0.63	0.13	0.77
LPA	0.59	0.14	

**Fig. 2:**
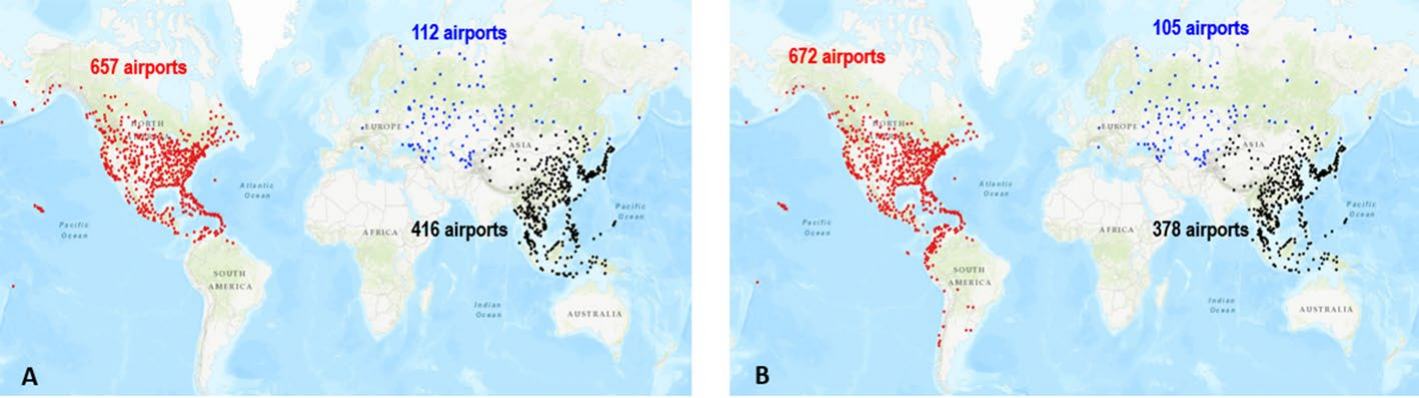
The community detection algorithms uncover similar communities. **a** Louvain reveals 3 communities in North and Central America-Caribbean (airports in red), East-Southeast Asia (airport in black), and in Russia-Central Asia-Transcaucasia (airports in blue). **b** LPA finds similar communities in these areas

**Fig. 3:**
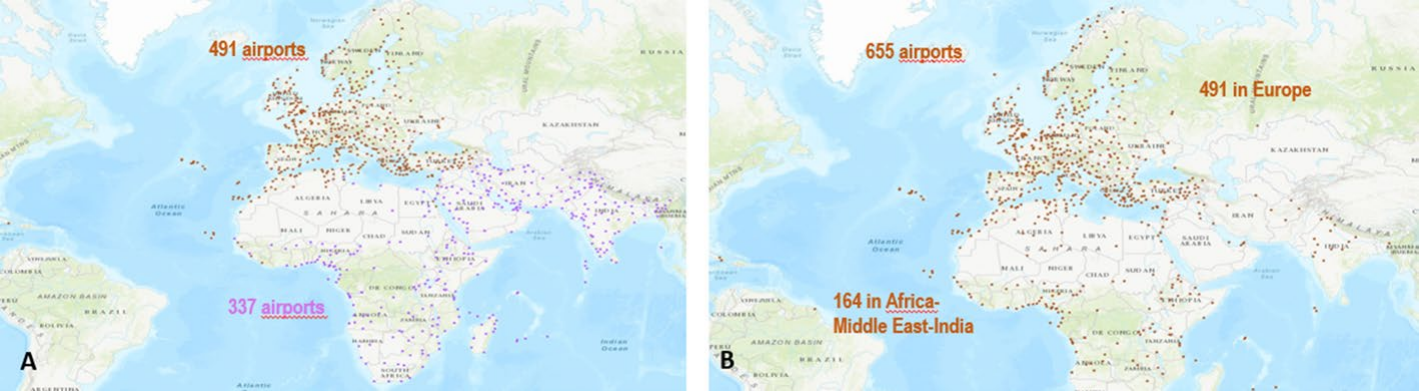
Communities uncovered by Louvain are grouped in a single community by LPA. **a** Louvain reveals two communities in Europe (airports in brown) and Africa-Middle East-Southern Asia (airports in lavender). **b** LPA finds a unique community covering the same geographical area

**Fig. 4:**
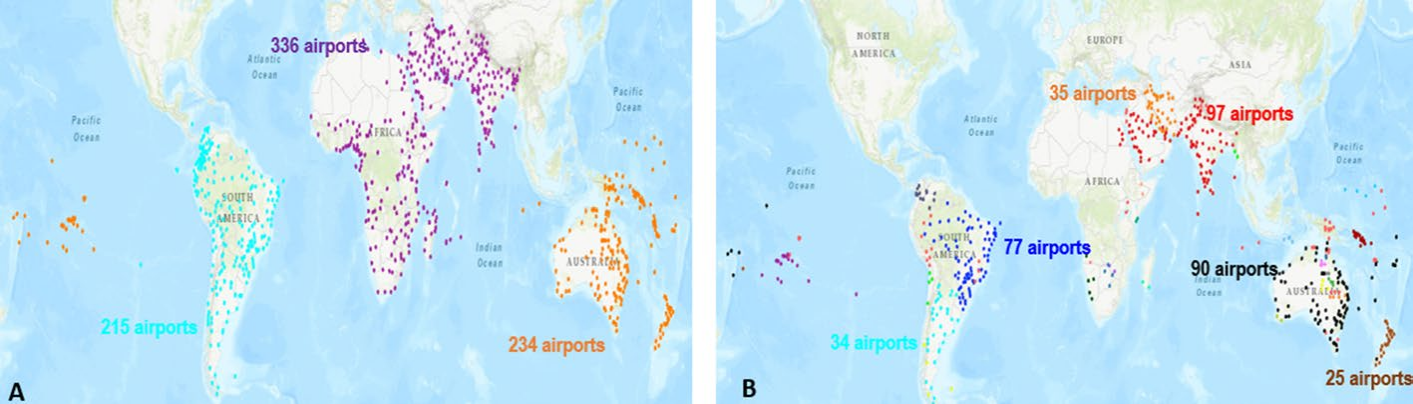
Large communities uncovered by Louvain split into multiple communities by LPA. **a** Louvain reveals 3 communities. The indigo points represent the airports located in Africa-Middle East-Southern Asia. The orange points represent the airports located in Oceania. The cyan points represent the airports in South America. **b** LPA uncovers 57 communities in the same geographical areas. There are 16 communities in Africa-Middle East-Southern Asia, 28 in Oceania, and 13 in South America

One can classify the communities uncovered by the community detection algorithms into two types. The first type corresponds to large size communities covering large geographical areas. Typically, these communities include several countries and contain hundreds of airports. The second type of community covers small geographical areas (within a country or fewer countries) and has less than one hundred airports. Louvain identifies seven large communities, and LPA discovers four large communities. Among these communities, three are of comparable size and cover the same geographical areas. Figure 2 represents the airports in these communities with the same colors. They are located respectively in North and Central America-Caribbean, East and Southeast Asia, and Russia-Central Asia-Transcaucasia. The fourth large community uncovered by LPA regroup into a single community, airports belonging to different communities uncovered by Louvain. Figure 3 illustrates this behavior. Indeed, parts of European (brown color) and Africa-Middle East-Southern Asia (lavender color) communities uncovered by Louvain merge in the same community (brown color) with LPA. Finally, the three other large communities uncovered by Louvain (Africa-Middle East India, South America, Oceania) split into multiple communities covering smaller geographical areas with LPA. Figure 4 illustrates this behavior. For example, LPA divides into 28 communities the South America (orange color) community of Louvain.

## Local component structure of the world air transportation network

This section reports a comparative analysis of the macroscopic topological properties of the local components. To this end, we use the community structure uncovered by the Louvain algorithm to extract the components. Louvain uncovers 27 communities. Consequently, there are 27 local components. One can classify them according to their size into two categories. The seven large local components contain more than 100 nodes, and the size of the 20 small local components ranges from 2 to 60. Large components cover ample geographical areas, including several countries, while small components are localized in a single country.

**Fig. 5:**
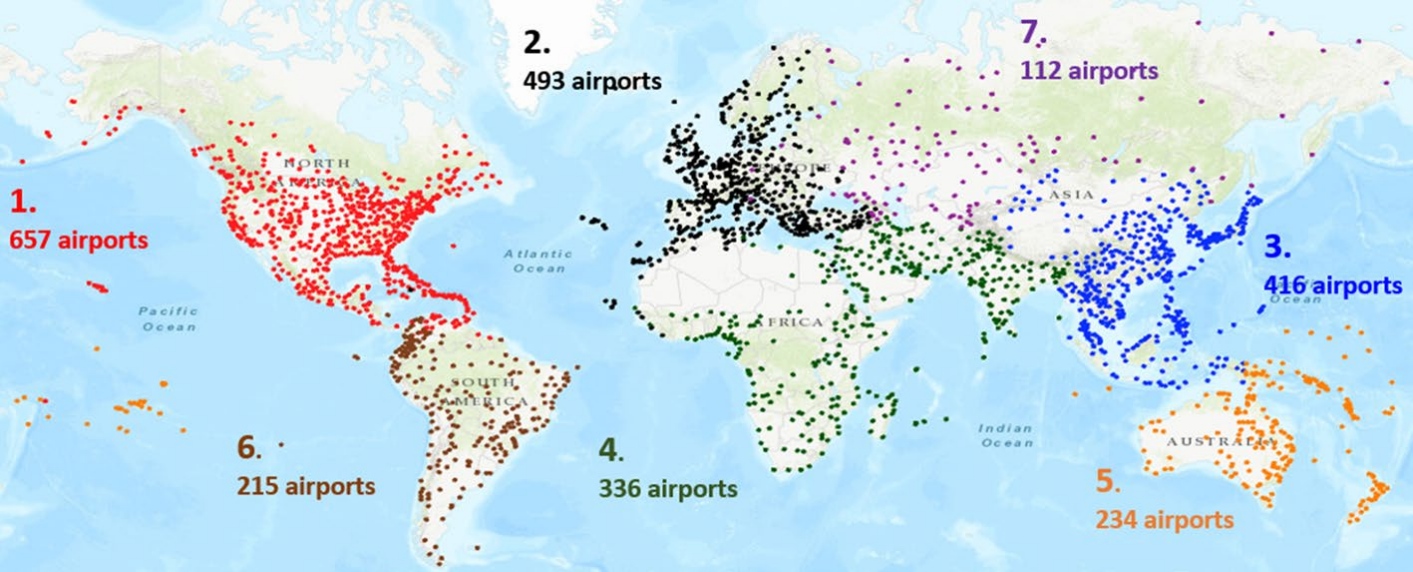
The seven large local components. (**1**) Airports in the North and Central America-Caribbean are in red. (**2**) Airports in the European component are in black. (**3**) Airports in the East and Southeast Asia component are in blue. (**4**) Airports in the Africa-Middle East and India component are in green. (**5**) Airports in the Oceania component are in orange. (**6**) Airports in the South America component are in brown. (**7**) Airports in the Russia-Central-Asia-Transcaucasia component are in indigo

Numerous schemes exist for partitioning the world into regions. The OAG divides it into seven regions: Europe, North America, Latin America, Africa, Asia, Middle East, Oceania. The subdivision corresponding to the local components is slightly different (see Fig. 5). Indeed, the large components cover the following regions: North-Central America-Caribbean (657 airports), Europe (493 airports), East-Southeast Asia (416 airports), Africa-Middle East-Southern Asia (336 airports), Oceania (234 airports), South America (215 airports) and Russia-Central Asia-Transcaucasia (112 airports). Altogether, they include 90% of airports in the world. Note that the component division does not reflect strict geographical divisions. It instead corresponds more to the political, cultural, historical, and economic divide. For example, some African airports located in Morocco and Tunisia belong to the European component because of the solid economic and historical ties these countries share with Europe.

**Fig. 6:**
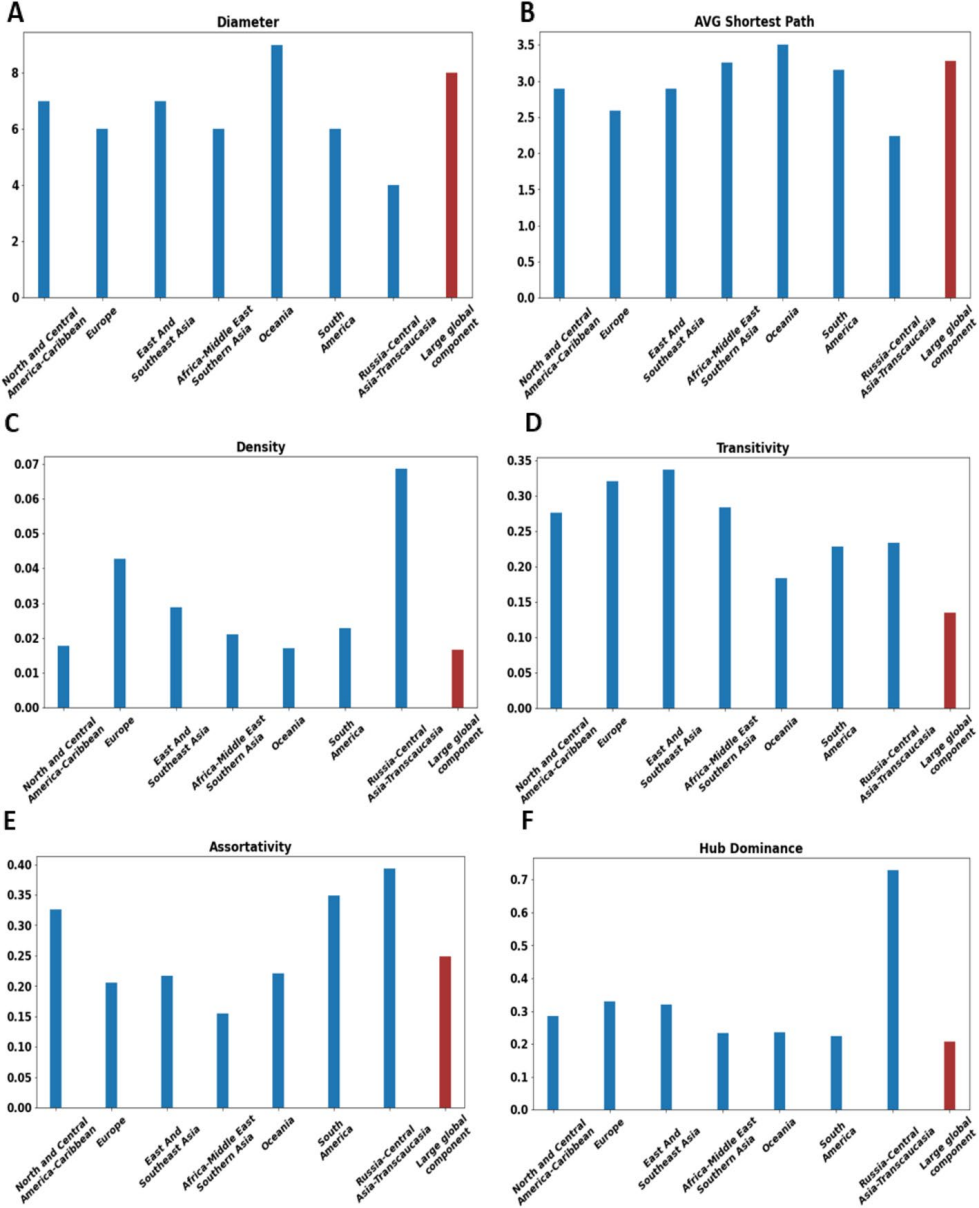
Basic topological properties of the large components. Each bar plot concerns a different property (Diameter, Average shortest path, Density, Transitivity, Assortativity, and Hub dominance). The blue bar corresponds to the large local component. The red bar corresponds to the large global component. For assortativity, absolute values are plotted, but in reality, the values are negative

### Analysis of the large local components

#### Basic macroscopic topological properties

The bar plots of Fig. 6 report the values of basic topological properties (diameter, average shortest path, density, transitivity, assortativity, hub-dominance) used to compare the structure of the large local components. The *shortest path length* between node *i* and node *j* is the minimal number of links between those two nodes. The *diameter* is the largest shortest path between any two nodes of the network. It informs us about the longest route. Results show that between six or seven flights are needed to reach the most distant airports in the large local components. It is not the case in the Oceania and Russia-Central Asia-Transcaucasia components. Indeed, in the Oceania component, a maximum of nine flights are required. Whereas in the Russia-Central Asia-Transcaucasia component, one reaches the most distant airports in four flights. Consequently, if we consider this worst-case scenario, it is better to travel in Russia-Central Asia-Transcaucasia, and one should avoid Oceania.

The *average shortest path length* indicates how many flights are needed on average to reach a destination. North- Central America - Caribbean and East-Southeast Asia components are comparable with around 2.8 flights. It is easier to travel in Europe with its average shortest path of 2,58. Destinations are easy to reach in the Russia-Central Asia-Transcaucasia component. Indeed, one needs a little bit more than two flights on average to reach his goal. One needs more flights to reach his destination on average in Oceania, Africa, Africa-Middle East-Southern Asia, South America. Indeed, the average shortest path ranges from 3,15 to 3,5 in these regions.

Let N the number of nodes and |*E*| the number of edges of a network. Its *density* $\mu$ measures the proportion of connected nodes as compared to the maximum number of possible connections. It is given by:

1 $$\begin{aligned} \mu = \frac{2*|E|}{N(N-1)} \end{aligned}$$

*Density* is a marker of easiness to travel. Ideally, one would like to reach any destination with one flight. However, local components are all sparse for economic reasons. This parameter highlights different situations. Russia-Central Asia-Transcaucasia ranks first with a density value of 0.068. Europe (0.042) and East-Southeast Asia (0.029) follow. For the remaining components, the gap broadens. Indeed, South America (0.022) and Africa-Middle East-Southern Asia (0.021) have comparable densities. Oceania and North- Central America–Caribbean components (0.017) are at the end of the scale.

*Transitivity* measures the proportion of triangles into a network. It refers to the probability that two nodes connected to a third one are also directly connected. It is given by:

2 $$\begin{aligned} \zeta = \frac{3* \text {number of triangles}}{\text {number of triplets}} \end{aligned}$$

*Transitivity* captures the local cohesiveness of a node. It reflects the easiness of reaching neighboring airports. The larger its value, the more easily passengers can be transferred to reach their destination if a direct flight becomes unavailable. From this point of view, Europe and East and Southeast Asia components are the most transitive, with around one-third of triangles. Transitivity is slightly below for the Africa-Middle East-Southern Asia (0.28) and North- Central America–Caribbean (0.27) components. One step beyond, Russia-Central Asia-Transcaucasia (0.23) and South America (0.22) present similar behavior. Finally, the Oceania component is the less cohesive, with 18% of triangles.

*Assortativity or degree correlation* indicates the mixing pattern of nodes. Assortativity value ranges between −1 and 1. If it is close to 1, nodes with a similar degree tend to be linked together. In this case, the network is assortative. Instead, if it is close to −1, low degree nodes tend to associate with high degree nodes. In this case, the network is disassortative. All the components are disassortative. This general pattern corroborates the tendency of hub-and-spoke organization, where a few interconnected hubs collect the traffic of peripherical low-degree destinations. Based on the observed values, we can form three groups. The first contains Russia-Central Asia-Transcaucasia, South America, North- Central America–Caribbean components with transitivity ranging from −0.39 to −0.32. The second group including Oceania, East, and Southeast Asia, and Europe, with values ranging from −0.22 to −0.20 exhibits a less pronounced disassortative pattern. Finally, Africa-Middle East-Southern Asia with a value of −0.15 has a more homogeneous mixing. Indeed, airline deregulation is not yet fully implemented for political and geographical reasons. Hub dominance represents the proportion of nodes connected to the highest hub within a community (Lancichinetti et al. 2010). In this work, we adopt the definition for the components. In this case, it represents the proportion of nodes connected to the largest hub within a component. It is computed as the ratio between the degree of the largest hub of the component *c* and the size of the component *c*. Its value ranges from 0 to 1. Let $k_i$, the degree of node *i*, $n_c$, the number of nodes of a component *c* and $K = \left\{ k_1, k_2,..., k_{n_c}\right\}$, the set of the nodes’ degrees. The component hub dominance is defined as follows.

3 $$\begin{aligned} \eta (c) = \frac{max(K)}{n_{c}} \end{aligned}$$

*Hub-dominance* allows identifying the level of centralization of the components. The higher the hub dominance, the more fragile the network to a targeted attack on its principal hub. We can distinguish three categories of components. The first category, the Russia-Central Asia-Transcaucasia component, is highly centralized. Indeed, the Domodedovo airport has a direct link with 73% of the airports. This ratio indicates over-centralization, with the majority of people from all over the region traveling through Moscow. This predominance is prejudicial to interregional relations. In the second category, the major hubs share direct flights with around one-third of airports in their components. Amsterdam, Beijing, and Atlanta international airports are those major hubs respectively in Europe, East, and Southeast Asia, and North- Central America – Caribbean components. In the last category formed by Africa-Middle East-Southern Asia, Oceania, and South America, the main hubs (Dubai, Sydney, and São Paulo) are less influential. They allow reaching directly around 20% of the destinations in their region.

*Small-world* networks have short path lengths and high clustering. It is a common property of numerous real-world networks. Instead, small path length and low clustering are characteristics of random networks. Table 3 reports the clustering coefficients and the average shortest path lengths of the seven large local components and their corresponding random networks. It shows that the clustering coefficients *C* of the components are significantly higher than random network values $C_{rand}$. Furthermore, the average shortest path length values *L* of the large local components are in the same order of magnitude as their corresponding random network $L_{rand}$. Therefore, one can conclude that large local components are small-world.

**Table 3:** Small-world property of the components

	***C***	***C***_***rand***_	***L***	***L***_***rand***_
North and Central America-Caribbean	0.491	0.0185	2.88	2.892
Europe	0.462	0.044	2.58	2.348
East and Southeast Asia	0.511	0.028	2.89	2.687
Africa-Middle East-Southern Asia	0.504	0.0187	3.25	3.178
Oceania	0.481	0.010	3.5	4.039
South America	0.359	0.015	3.15	3.528
Russia-Central Asia-Transcaucasia	0.495	0.069	2.23	2.531
Large global component	0.11	0.014	3.28	3.13
World air transportation network	0.46	0.005	3.86	3.45

*C* and $C_{rand}$ are respectively the clustering coefficients of the local components and the random network with same number of nodes and links. *L* and $L_{rand}$ are respectively the average shortest path length of the components and their corresponding random network

#### Degree distribution

**Fig. 7:**
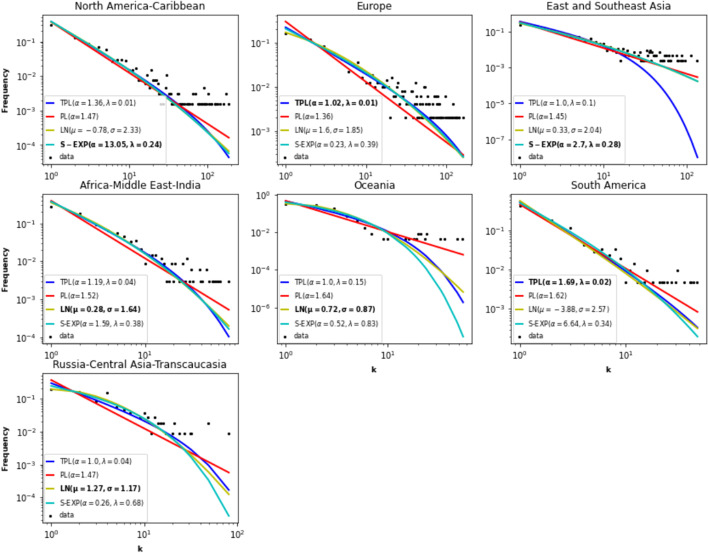
Degree distributions of the large local components. Empirical distribution (**dot**), estimates (**line**).The distributions under test are: power law (PL), truncated power law (TPL), log-normal (LN) and stretched exponential (S-EXP). The values in bold are the parameters of the best fit according to the Kolmogorov–Smirnov test

The degree of a node corresponds to its number of links. The *degree distribution* *P*(*k*) of a network is estimated using the fraction of nodes in the network with degree *k*. The *degree distribution* of many real-world networks is generally well-approximated by a power law ($P(k) = k^{-\alpha }$, where $\alpha$ is a positive exponent). Figure 7 represents the empirical degree distribution and four density estimates (power-law, truncated power-law, log-normal, stretched exponential) of the large local components. The power law possesses a single parameter, while its alternatives have two degrees of freedom. Table 4 reports the values of the Kolmogorov-Smirnov (KS test) goodness of fit test. The smaller the value, the better the fit. Note that the two-parameters density functions outperform the power-law in any case. These results corroborate previous studies (Broido and Clauset 2019). Indeed, the power law is a good fit for a wide range of degrees before deviating for large degrees. One can explain this phenomenon with the cost of adding new destinations. Indeed, the preferential attachment mechanism, typical of scale-free networks, where new nodes connect preferentially to highly connected nodes, tends to weaken. Due to space and time constraints, highly connected airports tend to limit their connections. Therefore, the distributions with two parameters distributions better approximate this behavior. Note that none of the distribution under test is a better fit for all the local components. Whatever, they are very similar, and they are all characterized by heavy-tails.

If we refer to the power-law exponent, it seems that the large local components exhibit a hub-and-spoke configuration. Three categories emerge. The first category with the smallest exponent value concerns the European component. As it includes the most significant proportion of hubs, it is the one with the less pronounced hub-and-spoke structure. The North and Central America-Caribbean, East and Southeast Asia, Africa-Middle East-Southern Asia, and Russia-Central Asia-Transcaucasia components form the second category. These components have almost the same proportion of hubs. Finally, the last category includes the Oceania and South America components. These components have the smallest proportion of hubs. The vast majority of the air traffic goes through these hubs. Thus, these components are more hub-and-spoke. Remember that the hub-and-spoke configuration is economically efficient, but it is more vulnerable to targeted attacks.

**Table 4:** Kolmogorov–Smirnov goodness of fit values (KS-test) for the degree distribution of the components

	Power law	Truncated power paw	Log-normal	Stretched exponential
North and Central America-Caribbean	0.073	0.012	0.011	**0.01**
Europe	0.136	**0.024**	0.038	0.032
East and Southeast Asia	0.105	0.157	0.025	**0.023**
Africa-Middle East-Southern Asia	0.111	0.021	**0.012**	0.015
Oceania	0.150	0.078	**0.050**	0.056
South America	0.061	**0.014**	0.015	0.027
Russia-Central Asia-Transcaucasia	0.176	0.051	**0.033**	0.035
Global component	0.103	0.025	**0.024**	0.123

Distributions under test are power law, truncated power law, log-normal, and stretched exponential. The smallest value (in bold) corresponds to the best fit

#### Degree-degree distance distribution of the local components

The power law assertion for the degree distribution is controversial (Broido and Clauset 2019; Voitalov et al. 2019). Thus, the authors of Zhou et al. (2020) propose to characterize real-world networks using the degree-degree distance $\eta$, $\eta (i,j) = max \left\{ k_{i}, k_{j}\right\} /min\left\{ k_{i}, k_{j}\right\}$. $\eta$ is defined for each link. They show that for many real-world networks, the power law is a better fit for the degree-degree distance distribution as compared to the degree distribution. This feature is more pronounced for dense networks.

**Fig. 8:**
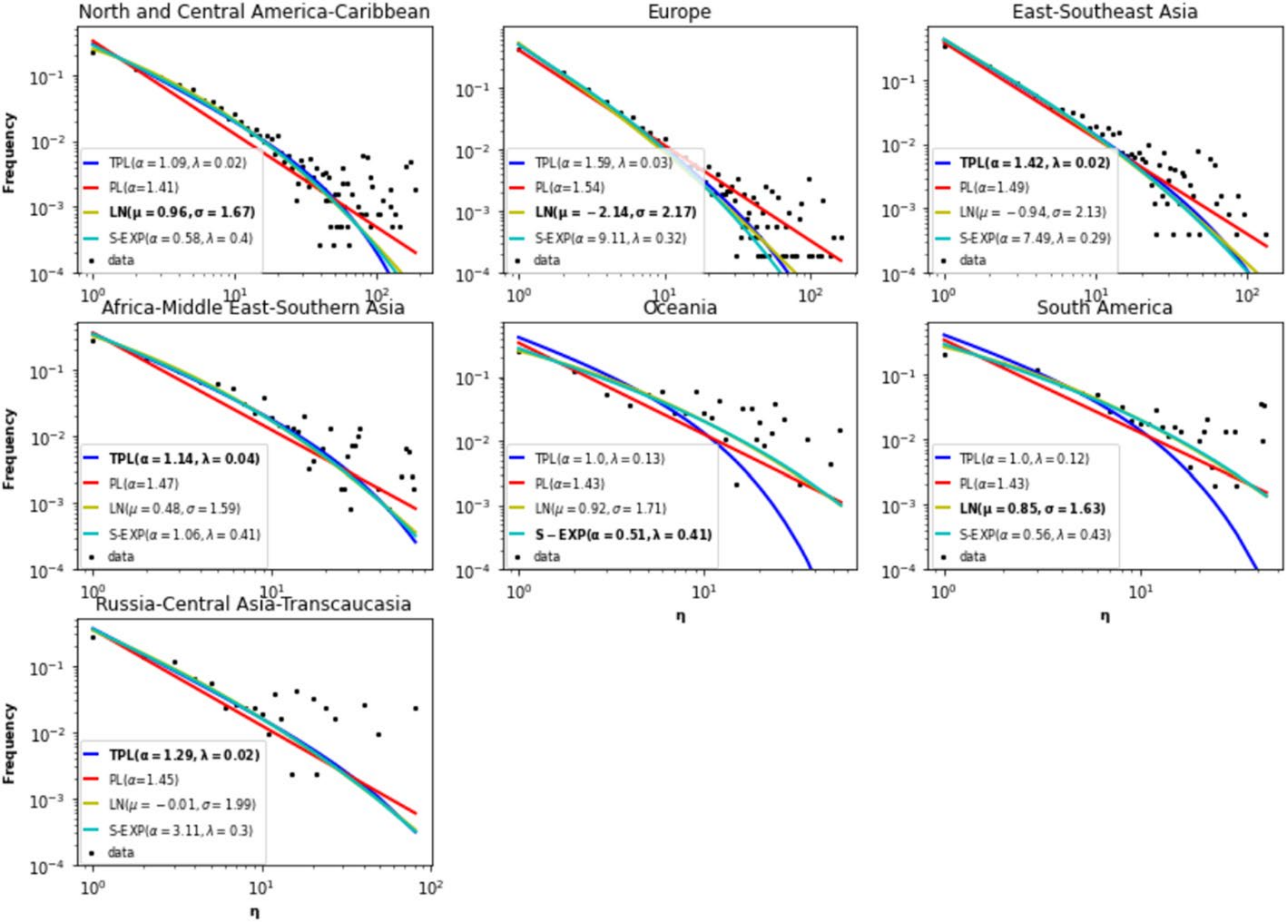
Degree-degree distance distributions of the components. Empirical distribution (**dot**), estimates (**line**).The distributions under test are: power law, truncated power law, log-normal and stretched exponential. The values in bold are the parameters of the best fit according to the Kolmogorov-Smirnov test

The degree-degree distance distribution of each large component is estimated. The results reported in Fig. 8 show that this distribution describes better the scale-free property of the components. Indeed, for three components (East and Southeast Asia, Africa-Middle East-Southern Asia and Russia-Central Asia-Transcaucasia), their degree-degree distance distribution follows a truncated power law, which is a power law with a cutoff. Whereas, the degree distribution of two components follow a truncated power law. The degree-degree distance of the North and Central America-Caribbean, Europe, and South America components can be better modeled by a Log-Normal law. In the Oceania component, the degree-degree distance distribution follows a stretched exponential (Table 5).

**Table 5:** Kolmogorov–Smirnov goodness of fit values (KS-test) for the degree-degree distance distribution.

	Power law	Truncated power law	Log-normal	Stretched exponential
North and Central America-Caribbean	0.117	0.021	**0.009**	0.014
Europe	0.091	0.013	**0.01**	0.025
East and Southeast Asia	0.101	**0.012**	0.019	0.022
Africa-Middle East-Southern Asia	0.127	**0.013**	0.022	0.017
Oceania	0.146	0.282	0.060	**0.054**
South America	0.136	0.190	**0.025**	0.030
Russia-Central Asia-Transcaucasia	0.103	**0.022**	0.025	0.024
Global component	0.103	**0.022**	0.025	0.024

Distributions under test are power law, truncated power law, log-normal, and stretched exponential. The smallest value (in bold) corresponds to the best fit

#### Distribution of airports by country

**Fig. 9:**
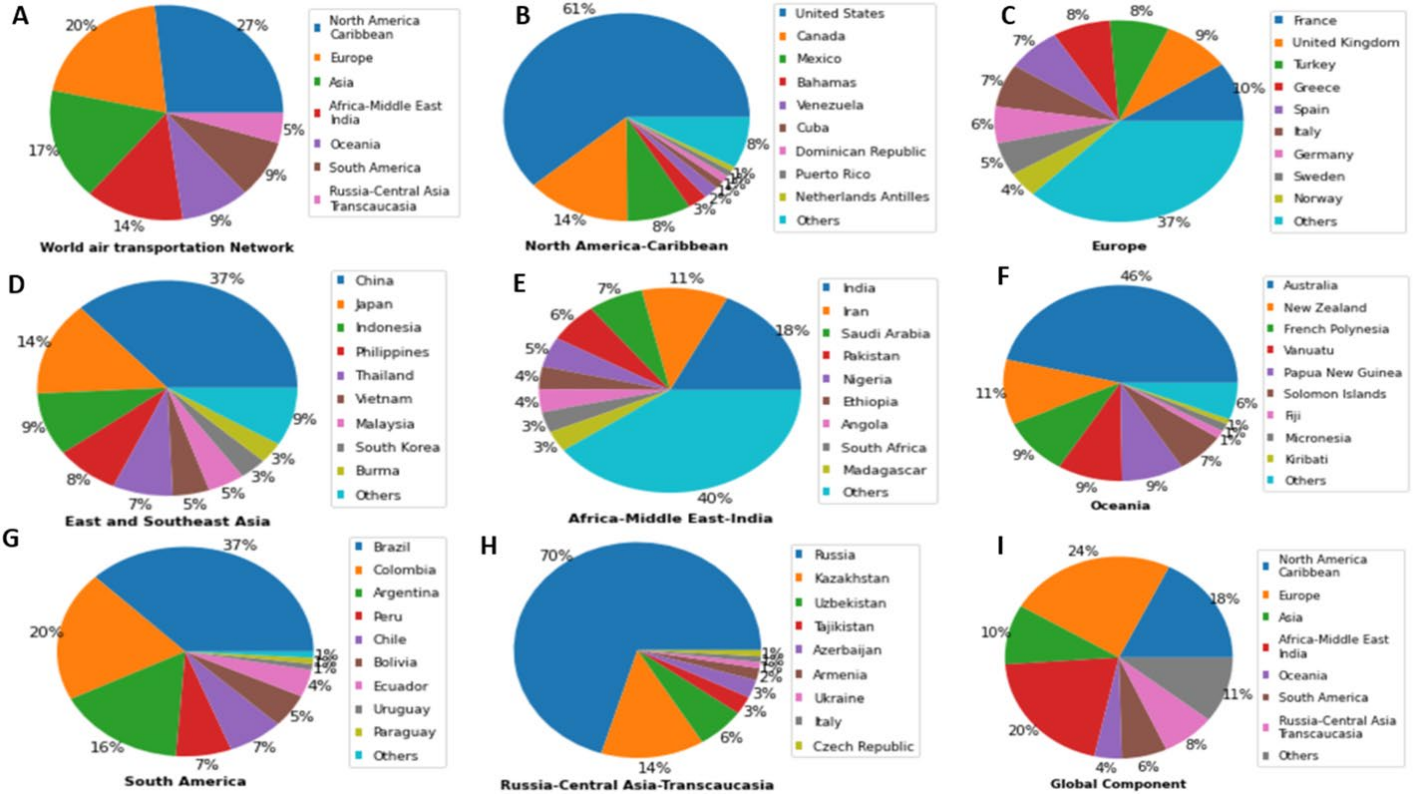
Figure **a** represents the proportion of airports in each main local component of the world air transportation network. Figures **b**–**h** reports the distribution of airports in the various countries of the seven large local components. **i** presents the distribution of airports per country in the large global component

Figure 9 A illustrates the distribution of the number of airports across the large local components. One can see that they are a bit unevenly distributed. Indeed, the three most significant components (North-central America-Caribbean, Europe, East-Southeast Asia) contains almost two-thirds of the airports (64%). The Fig. 9b–h report the distribution of airports by country in the seven large local components. We can distinguish three typical cases. In the first case, one country predominates heavily. It is the case in the North America-Caribbean component. Indeed, 61% of the airports are located in the United States. It is followed by Canada, with 14% of the airports. The remaining countries have a share far below 10%. The same situation characterizes the Russia-Central Asia-Transcaucasia component. Russia has 70% of the airports. Kazakhstan comes in the second position with 14% of airports.

In the second case, the gap between countries is less pronounced. (See Fig. 9d,f,g). It includes the East and Southeast Asia, Oceania, and South America components. In these components, the two first countries share more than 50% of the airports, and the others generally contain less than 10%. For instance, in the East and Southeast Asia components, China has the largest ratio of airports (37%), followed by Japan (14%). Australia (46%) and New Zealand (14%) dominate in Oceania. Brazil (37%), Colombia (20%), and Argentina (16%) dominate in South America.

The last case concerns the European and Africa-Middle East-Southern Asia components (See Fig. 9c, e). In these components, the proportion of airports by country is more evenly distributed. For instance, France covers 10% of airports, and the United Kingdom has 9% of airports in the European component. In Africa-Middle East-Southern Asia, 18% of airports are in India and 11% in Iran.

### Analysis of the small local components

**Fig. 10:**
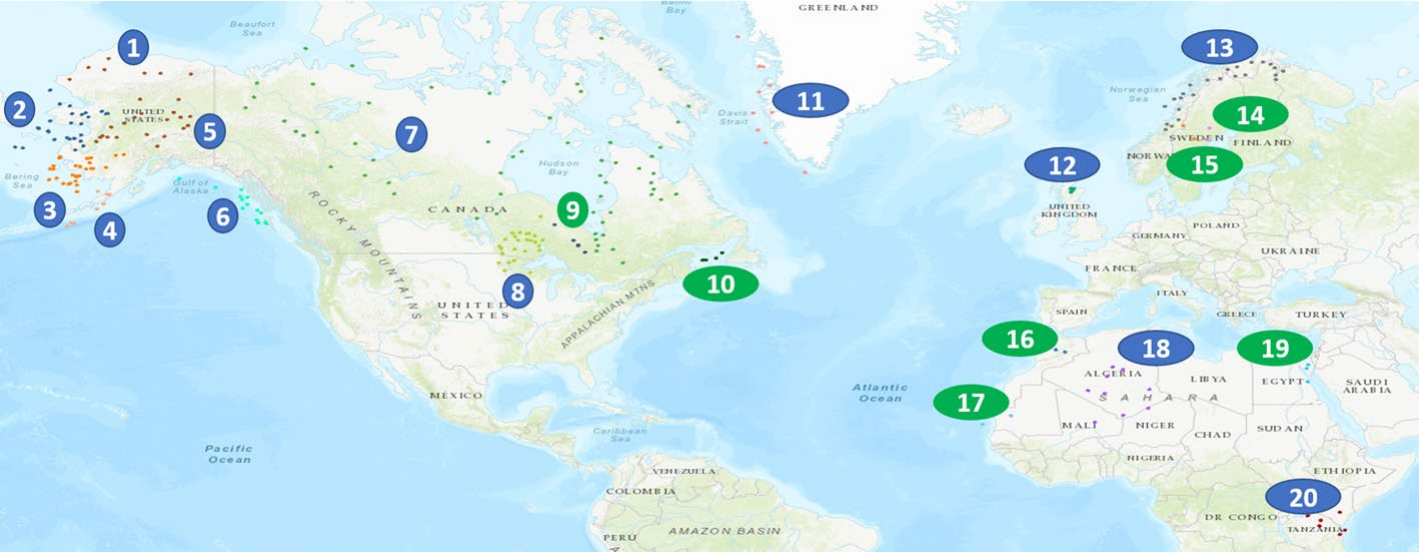
The twenty small local components. The blue circles show the significant small components (size greater than five airports). The green circles show the small components with sizes up to five airports. The geographical areas not appearing in the figure do not contain small components

Figure 10 presents the airports included in the twenty small local components. They group airports from a single country or state. Among these components scattered in North and Central America-Caribbean, Europe, and Africa-Middle East-Southern Asia areas, nine contain less than five airports (3 in Canada, 3 in Europe, 2 in Africa). In the following, we consider only significant small components containing more than five airports in the analysis.

Alaska possesses five significant small local components. Three of comparable size serving around thirty airports and two with fourteen and eight airports. As many towns cannot be reached by road, air transportation is well-developed, leading to the small local component organization in the different regions of Alaska. One to three airports in each component receives flights from outside and serves cities located inside the component. For example, the Fairbanks Airport serves the majority of the airports in its component (See Fig. 17 in appendix).

Canada contains two significant small components. The largest one connects 60 airports in the three territories: Northwest, Yukon, Nunavut.In these areas, cities are mainly reachable by plane due to climatic conditions and lack of road infrastructure. This component is well-connected with the rest of the country. Indeed, 12 airports share external liaisons with 17 airports outside the component. The second small component located in Ontario contains 25 airports. This star-shaped component is centered in Sioux Lookout, “The Hub of the North.” It is reachable by three airports (Thunder Bay Airport, Red Lake Airport, and Kenora Airport) (see Fig. 17 in appendix).

Europe has three significant small local components in Norway, Green-Land, and Scotland, with respectively 25, 10, and 7 airports. Located in northern regions, they are not easy to reach by other modes of transportation. The component in Norway is well-connected to the rest of the network, with ten airports sharing 23 liaisons with airports in other components. The Scottish component covers Orkney, an archipelago to the north of mainland Scotland. Only the airport of the largest city (Kirkwall) allows reaching this star-shaped component. Finally, one can attain the Green Land small local component that relies heavily on air transportation through two major airports (Kangerlussuaq and Godthaab/Nuuk) (see Fig. 17 in appendix).

Finally, in Africa-Middle East-Southern Asia, the most significant small local components are in Algeria (9 airports) and Kenya (6 airports). In Algeria, these airports, located in the Sahara, serve tourist and oil-producing areas. The Tamanrasset and Ouargla airports connect the other airports of this component. The airports that are in the small local component in Kenya regroup touristic venue. The Nairobi Wilson Airport is the center of this star-shaped component (see Fig. 17 in appendix).

#### Basic macroscopic topological properties

Table 6 reports the basic characteristics of the small components and the average shortest path length and transitivity of equivalent random networks. Note that we do not estimate the degree distribution because the components size is too small.

The *diameter* of the most significant small component in Canada is high. Indeed, it takes twelve flights to travel between the most distant airports. Diameter is also high for the small components located in the USA, Canada, Norway, Green Land, and Algeria. It ranges from 6 to 4. Travel is easier in the Scotland component with a diameter of 2, and Kenya with at most three flights to reach the most distant destinations.

One can distinguish three categories of components based on the *average shortest path lenght* values. The first category contains the largest small local component, covering the Canadian territories. An average of five jumps is necessary to join any two airports. The second category regroups the components in Alaska, Ontario, and Norway. With an average shortest path length ranging from 2,8 to 2,25, one reaches its destination on average with less than three flights. Although destinations are national, it is pretty efficient. Indeed, the shortest path length values are comparable to the large local components. The third category contains Green land, Scotland, Algeria, and Kenya. With an average shortest path length ranging between 2 and 1,47, one needs no more than two flights to reach any destination on average in these star-based components.

*Density* shows that it is generally more challenging to get a direct flight for a destination in the small local components than in the large ones. Indeed, the components are sparse. It is particularly true for the components located in Canada, Alaska, and Norway. Density is much higher for the Green Land, Algeria, and Kenya components. The one located in Scotland is the densest.

Overall, *Transitivity* is very low. Small components in Algeria and Kenya have no triplets. In Alaska and Canada, their fraction of triangles is lower than 10%. In Europe (Scotland, Norway, Green Land), small components are more transitive. Indeed, the degree correlation values range from 30% to 50%.

The small local components are generally more *disassortative* than the large ones. Indeed, their degree correlation coefficient varies from −0.75 to −0.38. Canada’s most significant small component is the only one deviating from this behavior with a much smaller value of −0.18.

A large *hub dominance* is a common feature of the vast majority of the small local components. The values ranging from 0.5 to 1 are characteristic of a star-based topology. Once again, Canada’s most significant small component departs from this typical behavior. Indeed, with a hub dominance value equal to 0.18, its topology is more string-based.

**Table 6:** Basic topological properties of the significant small local components (size greater than five airports)

Location	***N***	|***E***|	***d***	***L***	***L***_***rand***_	***C***	***C***_***rand***_	***μ***	***ζ***	***λ***	***η***
Canada	60	80	12	5	3.77	0.28	0.05	0.045	0.2	$-$0.18	0.18
Canada	25	27	5	2.45	3.38	0.17	0.108	0.09	0.056	$-$0.57	0.7
Alaska	29	33	6	2.8	3.20	0.2	0.065	0.08	0.07	$-$0.56	0.68
Alaska	27	37	5	2.5	3.2	0.45	0.037	0.11	0.17	$-$0.38	0.62
Alaska	27	40	4	2.4	2.80	0.6	0.078	0.11	0.24	$-$0.44	0.54
Alaska	14	15	4	2,25	2.74	0.08	0.217	0.16	0.063	$-$0.63	0.62
Alaska	8	6	3	0.6	2.35	0.04	0.112	0.21	0	$-$0.75	0.5
Norway	25	49	4	2.37	2.37	0.68	0.142	0.16	0.39	$-$0.43	0.5
Greenland	10	14	4	2	2.08	0.49	0.229	0.31	0.38	$-$0.4	0.67
Scotland	7	11	2	1.47	1.57	0	0.523	0.52	0.5	$-$0.47	1
Algeria	9	10	4	2	1.78	0	0.33	0.28	0	$-$0.73	0.5
Kenya	6	5	3	1.86	2.06	0	0	0.33	0	$-$0.74	0.8

*N* is the network size. |*E*| is the number of edges. *d* is the diameter. *L* is the average shortest path length. $L_{rand}$ and $C_{rand}$ are respectively the average shortest path and the average clustering coefficient of the corresponding random network. $\mu$ is the density. $\zeta$ is the transitivity. $\lambda$ is the assortativity also called degree correlation coefficient. $\eta$ is the hub dominance

**Fig. 11:**
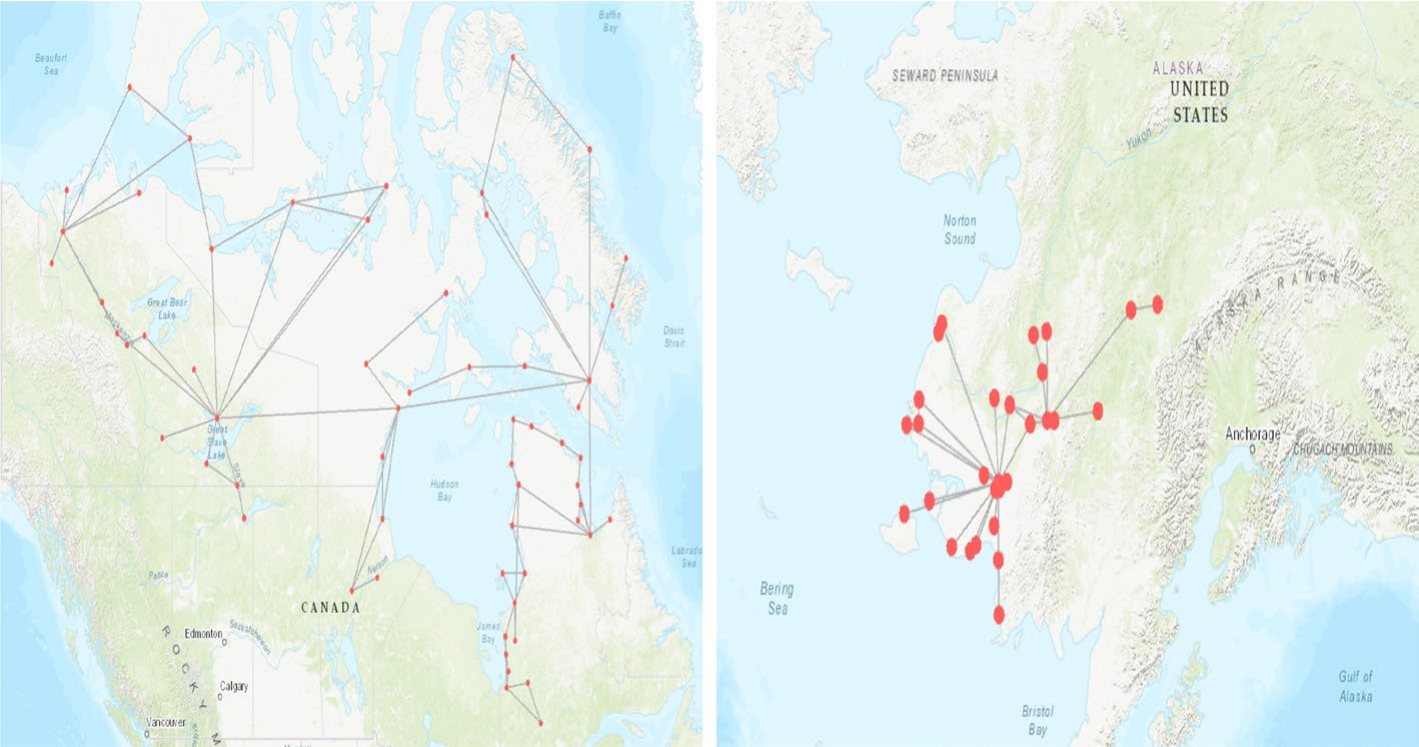
Typical small local components structures. The left image presents an example of string-based topology. It is the largest small local component in Canada. The right image gives an example of star-based topology. It is the second largest small local component in Alaska

Figure 11 illustrates the differences between these two topological organizations. In the string-based topology, the most connected nodes have almost the same degree. In contrast, a few hubs can reach more than half of the nodes in the star-based topology. Hubs in small components are gateways between other small airports and between large airports and small airports.

Five small local components out of twelve share the *small-world* property. Two in Europe (Norway, Greenland) and three in Alaska. Indeed, they have a similar or lower average shortest path than a random network. Additionally, their clustering coefficient is higher than in similar random networks. The largest component located in Canada is not small-world because of its high average shortest path length. The other components exhibit a low clustering coefficient compared to random networks.

To summarize, overall, small components appear in remote areas. They are usually poorly connected, making travels uneasy in these distant areas of the mainstream flows of travelers. They exhibit low transitivity and are highly disassortative. Their topology is usually star-based and more rarely string-based. They can be more or less connected to the main local component of their region.

## Global component structure of the world air transportation network

**Fig. 12:**
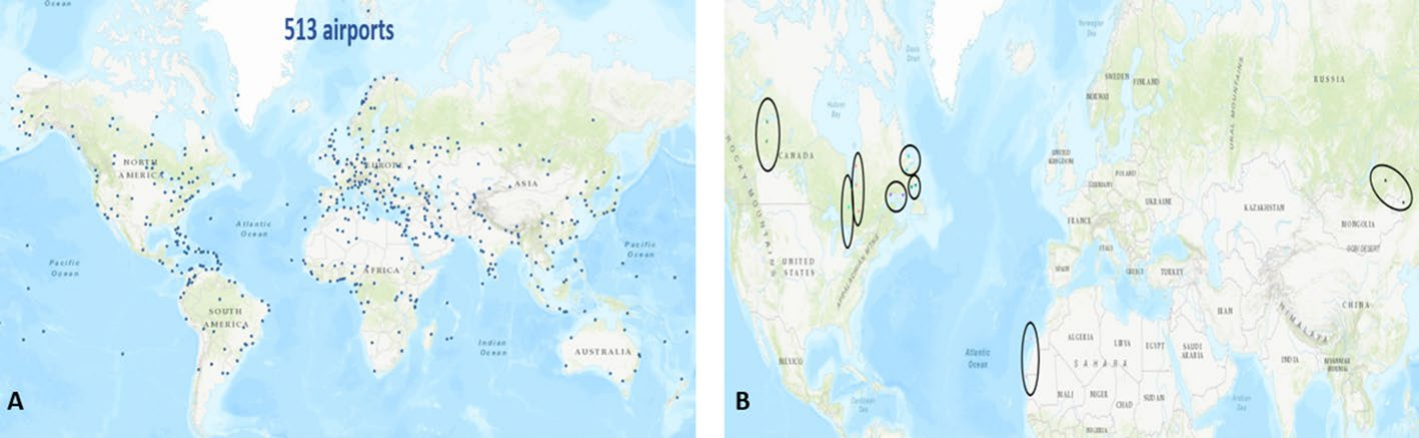
**a** The airports in the large global component. **b** The 8 small global components which are circled. The size range are between 2 and 3 airports

The global components contain the airports serving destinations outside their local components through their inter-regional links. There are one large and eight small global components. Figure 12 represents these components. One can notice that the large global component contains 513 airports covering the world. Most of the small global components are in the North and Central America-Caribbean area. Except for one of them, which contains three airports, the other components contain only two airports. One can see in Table 19 reported in the appendix, that 6 of them link airports in Canada included in the large North-Central America-Caribbean with close airports from small local components. The two others link two airports at the periphery of their component. Therefore, we analyze the large global component that we call global component in the following for short.

### Analysis of the large global component

Almost 20% of the airports in the world contribute to the global component. This significant proportion highlights the vitality of inter-regional exchanges. Its size (513 nodes) is in between the size of the European (493 nodes) and the North-Central America-Caribbean (657 nodes) large local components. In contrast, the number of links is much lower (2194). It compares to the number of links to East-Southeast Asia (2495) local components, which contains 20% fewer airports (416). Therefore, we focus the comparative analysis on these three local components.

#### Basic macroscopic topological properties

The *diameter* of the global component indicates that one needs a maximum of eight hops to join two airports. It is one flight more than in the North-Central America-Caribbean and East-Southeast Asia large local components, and two more flights compared with the local component of Europe.

The *average shortest path lenght* indicates that it is not as easy to travel in this component. Indeed, one needs more than three flights on average compared with less than three flights for Europe, North-Central America-Caribbean, and East-Southeast Asia local components. This parameter value is similar to the one observed in the Africa-Middle East-Southern Asia and South America local components.

*Density* values indicate that it is as challenging to travel in the global component as it is in North-Central America-Caribbean local component.

*Transitivity* is very low. Indeed, the fraction of triangles is 13%. It is almost two times less than in North-Central America-Caribbean component, and three times less than in the most transitive local component (East-Southeast Asia). Even Oceania, which is the less transitive local component, contains 18% of triangles. This result is expected. Indeed, it is more difficult to find an alternative to reach a destination in inter-regional routes often served by long-hauls.

The component is *disassortative*. Its degree correlation coefficient is comparable with that of the European, the East and Southeast Asia, and the Oceania components.

It has the lowest *hub-dominance* compared to the large local components. Indeed, the most prominent hub (Frankfurt Airport) allows reaching only 20% of airports in the components. This value is slightly lower than in the Africa-Middle East-Southern Asia, Oceania, and South America local components

Table 3 indicates that it is *a small world*. Indeed, its average path length is in the same order of magnitude as the equivalent random network. Furthermore, its clustering coefficient, even low, is ten times higher than for an equivalent random network.

These results are pretty interesting. They indicate that despite being not under the control of any particular airline, the inter-regional network is quite efficient. Indeed, it compares favorably with the regional component organization of the most advanced local components. Quite naturally, with minimum planning, it allows making worldwide travels almost as easy as regional travel. Being less centralized, it is also more resilient. Unfortunately, its lack of transitivity makes it difficult to use alternative routes.

#### Degree and degree-degree distance distribution

**Fig. 13:**
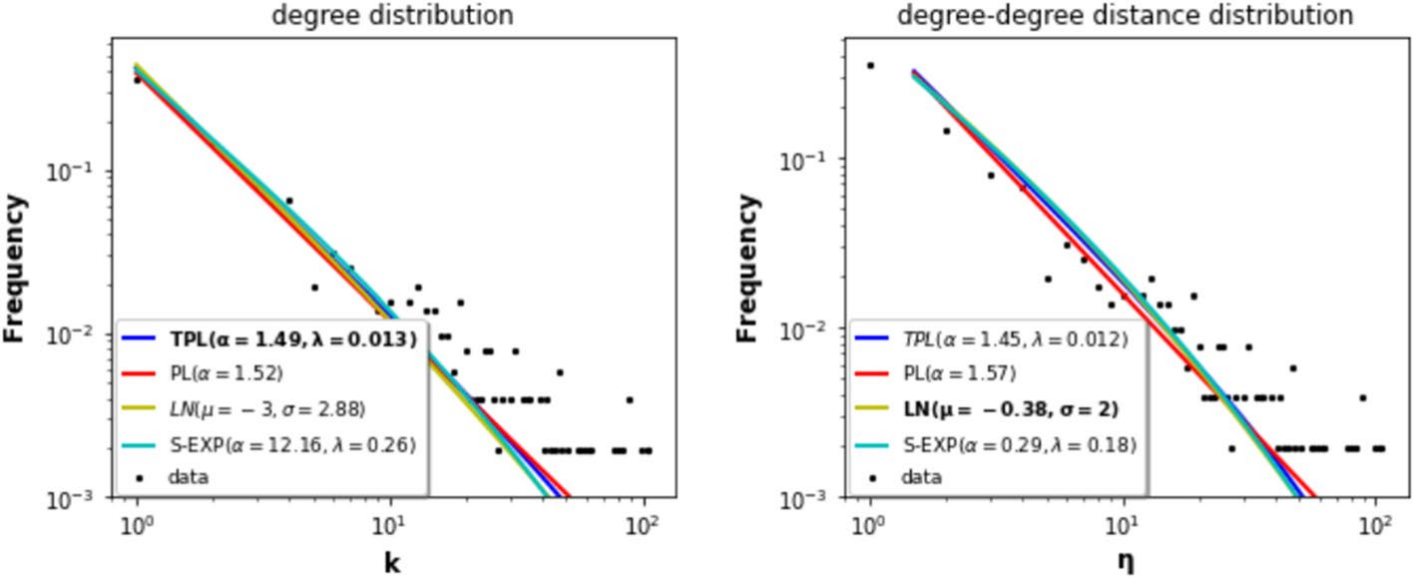
The degree (left) and degree-degree distance (right) distributions of the large global component. Empirical distribution (dot), estimates (line).The distribution under test are: power law (PL), truncated power law (TPL), log-normal (LN) and stretched exponential (S-EXP). The values in bold are the parameters of the best fit according to the Kolmogorov-Smirnov test

Figure 13 shows the degree and degree-degree distance distributions of the large global component. Visually, it is not so easy to point the differences between both distributions. According to the KS goodness of fit test, the truncated power law better approximates the degree distribution. The log-normal function is a better fit for the degree-degree distance distribution. Whatever, both distributions are heavy-tailed.

#### Distribution of the airports between regions

The pie-chart in Fig. 9i illustrates the distribution of the global component airports between the seven regions corresponding to the local components. Comparing this chart with Fig. 9a allows getting an idea about how the various regions are involved in the global air routes. One can see that these distributions, although correlated, can differ. Indeed, the regions with a big share of airports are not necessarily the most active in inter-regional traffic.

For example, the North and Central America-Caribbean local component contains the highest fraction of airports worldwide (27%). However, its share on the liaisons with the other regions is 18%. It indicates that a high proportion of airports serves local destinations. East and Southeast Asia exhibits a similar trend, with 17% of the airports in the world but only 10% of the inter-regional routes. It is also the case for South America and Oceania.

In contrast, Europe with its 20% of airports is more international with its 24% of airports exchanging flights with other regions. This behavior is more pronounced for Africa with its 14% of airports and 20% of inter-regional flights. It is probably due to political and historical reasons. Indeed, Africa still lacks global airlines. The air traffic flows mainly through Europe or the Middle East. Russia-Central Asia-Transcaucasia is intensely involved in inter-regional exchanges even if it has a low proportion of the world airports.

## Comparison of the world air transportation network with the large components

### Basic macroscopic topological properties

The basic topological properties of the world air transportation network are reported in Table 1.

The *diameter* of the world air transportation network is two to three times greater than the diameter of the large components. While the longer route ranges from 4 to 8 flights in the components, it takes 12 flights to join the furthest destinations worldwide.

Its *average shortest path lenght* is also higher compared to the components, but the difference is more moderate. Indeed, one needs on average 3,6 flights to reach any destination in the world compared to 3, 5 flights when traveling in the Oceania component.

*Density* shows that it is more uneasy to reach remote locations in the world air transportation networks than to travel in a single component. Indeed, the density is one degree of magnitude lower than in Europe

*Transitivity* is a good feature of the world air transportation network. With 26% of closed triangles, reaching a destination with a direct flight is as easy as traveling in the Africa-Middle East-Southern Asia component or the North-Central America-Caribbean component. Transitivity is higher than in the Oceania, South America, and Russia-Central Asia-Transcaucasia components.

It is slightly *disassortative*. Indeed, its degree correlation coefficient is approximately ten times higher in the local and global components.

It has the lowest *hub-dominance* compared to the components. Indeed, the largest hub (Frankfurt Airport) is linked to 9% of the airports worldwide. In the components, this value range from 20% to 73%.

It shares the *small world* property with the large components. Indeed, its average path length is low, and its transitivity is high compared to a similar random network, as indicated in Table 3.

### Degree and degree-degree distance distribution

**Fig. 14:**
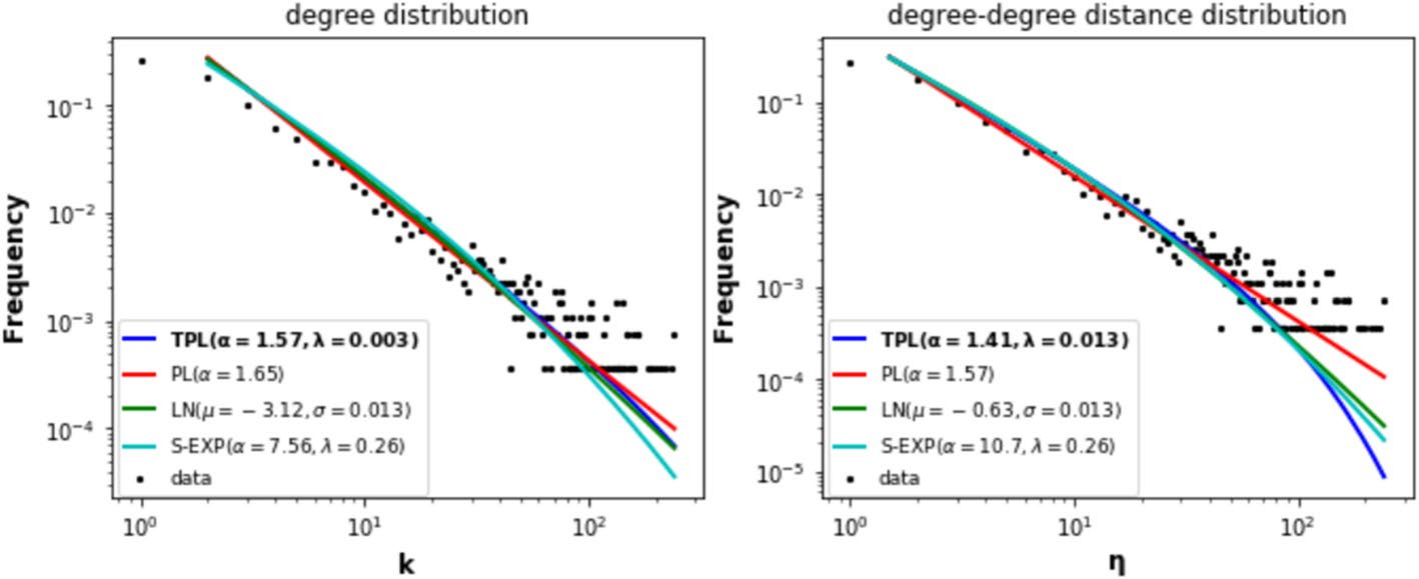
The degree (left) and degree-degree distance (right) distributions of the world air transportation network. Empirical distribution (dot), estimates (line). The distribution under test are: power law (PL), truncated power law (TPL), log-normal (LN) and stretched exponential (S-EXP). The values in bold are the parameters of the best fit according to the Kolmogorov-Smirnov test

Degree and degree-degree distance distributions of the world air transportation network are reported in Fig. 14. The truncated power law is the best fit in both cases. This result is in line with previous studies.

Overall, these results are very informative. They demonstrate the advantages of the component structure representation to get a clear picture of the diversity of the world air transportation network. Indeed, looking at the topological properties of the global air transportation network can create an inaccurate view of the actual situation. The regional and inter-regional situations are very diverse. Unfortunately, the network analysis offers an “average view” of a non-homogeneous world. Therefore, one needs to interpret these data very carefully.

## Degree centrality analysis

Centrality measures aim to assess the importance of a node based on some topological features. Degree centrality is among the most commonly used measure. It measures the node influence with its number of links. This section investigates the degree centrality to assess the influential airports according to the different views carried by the component structure. First, we consider the large local components to uncover the top connected airports at the regional level. Then, we use the large global component to rank the inter-regional airports. Finally, we compare these results to the top connected airports in the world air transportation network. This process allows us to highlight the differences between these three levels of analysis.

**Table 7:** Degree Centrality in the seven large local components

Large Local Component	Airport	City	Country	ID	LR	CP (%)
North and central America Caribbean	Hartsfield J	Atlanta	USA	187	1	23.89
	Dallas Fort Worth	Dallas	USA	172	2	35.92
	Chicago O’Hare	Chicago	USA	172	3	40.18
	Denver	Denver	USA	161	4	47.64
	George B Houston	Houston	USA	146	5	49.77
Europe	Schiphol	Amsterdam	Netherlands	162	1	32.86
	Munich	Munich	Germany	145	2	40.36
	London Stansted	London	UK	142	3	51.72
	Barcelona	Barcelona	Spain	137	4	55.78
	Frankfurt	Frankfurt	Germany	132	5	56.79
East and Southeast Asia	Beijing Capital	Beijing	China	133	1	31.97
	Guangzhou Baiyun	Guangzhou	China	112	2	37.25
	Shanghai Pudong	Shanghai	China	112	3	41.34
	Chengdu Shuangliu	Chengdu	China	95	4	44.47
	Taiwan Taoyuan	Taipei	Taiwan	87	5	46.63
Africa Middle East- Southern Asia	Dubai	Dubai	U A E	78	1	23.14
	King Abdudaziz	Jeddah	Saudi Arabia	63	2	30.56
	Indira Gandhi	Delhi	India	60	3	39.76
	Chhatrapati Shivaji	Mumbai	India	58	4	42.13
	Addis Ababa Bole	Addis Ababa	Ethiopia	56	5	51.33
Oceania	Sydney K Smith	Sydney	Australia	55	1	23.5
	Brisbane	Brisbane	Australia	48	2	31.19
	Auckland	Auckland	New Zealand	36	3	38.46
	Melbourne	Melbourne	Australia	33	4	40.59
	Port M Jacksons	Port Moresby	P New Guinea	24	5	48.71
South America	G G A F Montoro	Sao Paulo	Brazil	48	1	22.42
	El Dorado	Bogota	Colombia	44	2	40.65
	Viracopos	Campinas	Brazil	42	3	45.32
	Jorge Newbery	Buenos Aires	Argentina	41	4	60.28
	P J Kubistschek	Brasilia	Brazil	36	5	64.48
Russia Central Asia- Transcaucasia	Domodedovo	Moscow	Russia	81	1	72.32
	Pulkovo	St Petersburg	Russia	49	2	82.14
	Sheremetyevo	Moscow	Russia	49	2	84.82
	Tolmachevo	Novosibirsk	Russia	32	4	85.71
	Koltsovo	Yekaterinburg	Russia	31	5	85.71

The top five hubs for each large local component are reported. ID is the internal degree. It measures the number of connections of an airport with airports located in its large local component. LR is the airport local rank in its component by decreasing internal degree. CP is the cumulative fraction of connected airports represents the proportion of airports that the top *x* local hubs can reach in their components

### Exploring the regional hubs in the large local components

Table 7 lists the top 5 connected airports in the seven large local components called regional hubs. It also reports the cumulative proportion of airports connected to the regional hubs.

The five top connected airports of the *North and Central America-Caribbean component* are in the United States. They are in four different states (Illinois, Texas, Colorado, Georgia). They are the most important of major airlines in the United States. Indeed, each of these airports receives more or less 50 millions passengers per year. Hartsfield J Atlanta Airport is Delta Air Lines’ headquarter and one of its central hubs. It is one of the busiest airports worldwide, with almost one hundred millions passengers per year. The Dallas Fort Worth Airport ranks second. Located in Fort Worth, it is the primary hub of American Air Lines and Southwest Airlines. The third hub of this component, the Chicago O’Hare & Chicago Airport, is one of the main hubs of United Air Line (headquartered in Chicago) and American Air Line. Denver Airport is the fourth hub of this component. It has the largest area in the United States. Headquarter of the low-cost carrier Frontier, it is a crucial hub for United Air Lines. The second important airport in Texas, the George B Houston Airport, is this component’s fifth hub. It is one of the hubs of United Air Lines. Altogether, these first five hubs connect half of the airports in the component.

The top five hubs in the *European component* are in four different countries (Netherlands, Germany, UK, Spain). These airports are the stronghold of the biggest airlines in these countries. The number of passengers ranges from approximately 70 millions to 20 millions per year. The Amsterdam Schiphol Airport is the first hub of this component. It is the largest in the Netherlands and the most critical hub of KLM. It is also a very active platform for low-cost carriers such as EasyJet, Vueling, TUI Air Lines, Corendon Dutch. Munich Airport in Germany is second. It plays an essential role for Lufthansa Air Lines and its low-cost subsidiary (Air Dolomity, Eurowings) to serve European destinations. The London Stansted Airport in the United Kingdom is the third hub of the component. It is a major airport for low-cost carriers such as Ryanair, Pegasus, Easy Jet (Dobruszkes 2006). Due to its geographical position and the high level of tourism in Barcelona, its airport is very well connected. It receives about 20 millions passengers per year. Indeed, the Barcelona airport in Spain is the fourth most connected airport. It is the primary airport of the Vueling Air Lines. Frankfurt in Germany, the fifth hub, is the main historical airport of Lufthansa. While Munich is more focused on European destinations, Frankfurt is devoted to international destinations. These five hubs allow reaching about 56% of the airports of this component. The London Gatwick Airport (132 links) in the United Kingdom, Charles de Gaulle Airport (129 links) in France, Dublin Airport (128 links) in Ireland, Düsseldorf Airport (121 links) in Germany, and Ataturk Airport (121 links) are the five other hubs. The top first ten hubs can reach 66% of airports of the component. They are the home of the leading European carriers.

The largest four hubs in the *East and Southeast Asia component* are in different provinces and municipalities of China. The fifth hub is in Taiwan. These airports receive tens of millions of passengers per year. The most connected, Beijing Capital Airport, is one of the busiest airports in the world, with almost 100 millions passengers yearly. The Guangzhou Baiyun airport, the second hub of this component, is situated in the capital of the provincial Guangdong in South China. It is a hub of the China Southern Air Lines, which is headquartered in Guangzhou. The Shanghai Pudong Airport, situated in East Asia, has the same degree as the Guangzhou Baiyun airport. It is a major hub of the Shanghai and China Eastern Air Lines. The fourth hub, called Chengdu Shuangliu Airport, is in the capital of Sichuan province. Serving Central-western China, it is the central hub of the Sichuan and Chengdu Air Lines. The Taiwan Taoyuan Airport is the fifth regional hub in East and Southeast Asia. Serving Taipei, it is the largest airport on the island. Together, these hubs are connected to 46,6% of East and Southeast Asia airports.

The first five hubs of the *Africa-Middle East-Southern Asia component* span Africa, the Middle East, and Southern Asia. Indeed, two are in the Middle East, two in India and one in Africa. The first, Dubai airport, is one of the most important airports in the world. It can handle more or less 100 millions passengers. It is the most critical hub of Emirates Air Lines. King Abdulaziz Airport in Saudi Arabia ranks second. It is the largest airport in terms of surface area. Located near Mecca, it receives many visitors from different Muslim countries. The biggest airport in India, Indira Gandhi Airport, is third. Situated in the capital Delhi, a cosmopolitan and tourist city, it receives a lot of people. The Chhatrapati Shivaji Airport, located in the megacity of Bombay, the economic capital of India, is the following hub. It is the central hub of India Air Lines. The Addis Ababa Bole Airport in the capital of Ethiopia is the fifth hub of the component. Due to its political and geographical position, it connects Africa with the rest of the world. It is the hub of Ethiopia Air Lines, the first airline serving Africa’s airports. These five hubs connect about 51% of airports of the Africa-Middle East-Southern Asia component.

Among the top five hubs in the *Oceania component*, three are in Australia, one in New Zealand, and one in Papua New Guinea. These airports receive less than 50 millions passengers per year. The Sydney Kingsford Smith Airport is the largest hub of the Oceania component. Indeed, located in the capital city of New South Wales in Australia, Sydney is a metropolis and touristic city attracting people coming from different countries. It is the main hub of Qantas, the largest airline in Australia. The Brisbane Airport, situated in the capital city of Queensland, is the second most connected airport in this component. Like Sydney, Brisbane is also a tourist city that receives many people from different horizons. It is the primary airport of Virgin Australia and a hub for Qantas. The third hub, Auckland Airport, is the top airport in New Zealand and the base of Air New Zealand. The Melbourne-Tullamarine Airport located in Melbourne ranks fourth. It is essential for Jetstar Air Lines, based in Melbourne and Qantas. Melbourne is also a metropolitan city that receives numerous tourists. The fifth hub of this component, the Port Moresby Jacksons Airport, is the most influential airport of Papua New Guinea. Located near Port Moresby, this airport is the largest hub of Air Niugini, headquartered in Port Moresby. These five hubs serve 48,7% of the airports in the component.

Three of the top five hubs in the *South America component* are in Brazil, one in Colombia, and one in Argentina. Guarulhos G A F Montoro Airport, the first hub, is in São Paulo, the most populated city in Brazil. The busiest in Brazil (Couto et al. 2015), it is an essential airport for GOL (based in Rio de Janeiro) and ITA Air Lines (based in São Paulo). The second hub in Columbia, the El Dorado Airport, is in Bogotá, the capital of Colombia. It is the base for Avianca Air Lines. The Viracopos Airport in Campinas ranks third. It has a regional vocation in Brazil (Couto et al. 2015). It is a hub for Azul Brazilian Airlines, a low-cost airline. The Jorge Newbery Airport is the fourth hub of this component. Located in Buenos Aires, the capital of Argentina, it is a hub for the largest Argentina airline (Aerolíneas Argentinas). The fifth hub, the Presidente J Kubistschek, is in Brazilia, the capital of Brazil. It is one of the hubs of GOL and LATAM Brasil Air Lines. These five largest hubs reach around 64% of airports in this component.

In the *Russia-Central Asia-Transcaucasia component*, the top five airports are in the major cities of Russia. They receive less than 50 millions passengers per year. The Domodedovo Airport, located in Moscow, is the first hub. It centralizes about 72% of the routes. Sheremetyevo Airport in Moscow and Pulkovo Airport, the main airport of St Petersburg, are tied for second. Together the two top airports in Moscow concentrate 80% of the destination of the component. Therefore, Moscow is the heart of the component and the Russian air network in particular. This result corroborates the findings reported in Tarkhov (2017). Indeed, this paper shows that since the fall of the Soviet Union, the Russian air network is more and more centralized in Moscow. Almost all flights (regional and international) transit through this city. The Domodedovo Airport is a hub of the Domodedovo Air Lines, and the Sheremetyevo Airport is a hub of Aeroflot–Russian Airlines, the biggest in Russia. The Pulkovo Airport is the main airport of St Petersburg, the second-largest city in Russia. It is also an important airport for Rossiya Airlines. Another hub of this component is the Tolmachevo Airport, located near Russia’s major city, Novosibirsk. This airport has a regional vocation. It is an important airport for the Novaport Air Lines. Situated in Yekaterinburg city, the Koltsovo is the fifth most connected airport of this component. It is a vital hub for the Ural Air Lines, based in the same city as the airport. These hubs reach around 85% of airports in this component.

**Table 8:** Degree centrality in the large global component

Region	Airport	City	Country	ED	GRR	GRC
North and central America- Caribbean	John F Kennedy	New York	USA	62	1	9
	Lester B Pearson	Toronto	Canada	45	2	19
	Newark Liberty	Newark	USA	39	3	24
	Miami	Miami	USA	36	4	27
	Los Angeles	Los Angeles	USA	34	5	30
Europe	Frankfurt	Frankfurt	Germany	106	1	1
	Charles de Gaulle	Paris	France	104	2	2
	Atatürk	Istanbul	Turkey	99	3	3
	Heathrow	London	UK	89	4	4
	Schiphol	Amsterdam	Netherlands	80	5	7
East and Southeast Asia	Beijing Capital	Beijing	China	61	1	10
	Suvarnabhumi	Bangkok	Thailand	57	2	12
	Narita	Tokyo	Japan	56	3	13
	Incheon	Seoul	South Korea	48	4	15
	Hong Kong	Hong Kong	Hong Kong	47	5	16
Africa- Middle East- Southern Asia	Dubai	Dubai	U A E	89	1	4
	Hamad	Doha	Qatar	59	2	11
	Abu Dhabi	Abu Dhabi	U A E	42	3	22
	Cairo	Cairo	Egypt	34	4	30
	Indira Gandhi	Delhi	India	31	5	32
Oceania	Sydney K Smith	Sydney	Australia	26	1	41
	Melbourne	Melbourne	Australia	20	2	57
	Auckland	Auckland	New Zealand	13	3	96
	Perth	Perth	Australia	13	3	96
	Brisbane	Brisbane	Australia	12	4	106
South America	Guarulhos-G A F M	São Paulo	Brazil	36	1	26
	El Dorado	Bogotá	Colombia	30	2	36
	Jorge Chávez	Lima	Peru	22	3	53
	Ministro Pistarini	Buenos Aires	Argentina	19	4	61
	Rio G–T Jobim	Rio de Janeiro	Brazil	18	5	69
Russia- Central Asia- Transcaucasia	Sheremetyevo	Moscow	Russia	84	1	6
	Domodedovo	Moscow	Russia	77	2	8
	Pulkovo	St Petersburg	Russia	47	3	16
	Vnukovo	Moscow	Russia	31	4	32
	Heydar Aliyev	Baku	Azerbaijan	23	5	51

The top five inter-regional hubs in each region are reported. ED is the external degree. It measures the number of connections of an airport with airports located in the large global component. GRR is the global rank of the airport in its region. GRC is the global rank in the global component is the airport rank in the global component by decreasing external degree

### Exploring the inter-regional hubs in the large global component

We consider the large global component to extract the high degree airports. For comparative purposes, we classify the so-called inter-regional hubs in seven regions based on their local component. Indeed, an airport in a global component is also a member of a local component. Table 8 reports their coordinates and their external degree (number of links with airports outside their local component). Their global rank in the component is computed according to the decreasing order of their external degree. The global rank in their region measures their relative global position in their region. Four out of five of the top inter-regional hubs in the North and central American-Caribbean area are in the USA. These hubs are located on the coasts, closer to the other regions. The largest hub of this component is the John F Kennedy Airport, located in New York. This gateway to the United States is the busiest airport for international flights, with around fifty millions passengers yearly. It is an essential platform for American Airlines, Delta, and Jet Blue. The most important airport in Canada, the Lester B Pearson Airport, is the second-largest inter-regional hub. It connects Canada and the US to the other regions. Situated in Toronto, it is the primary hub of Air Canada. Newark Liberty Airport ranks third. This crucial hub of United Airlines serves the New York metropolitan area. Miami and Los Angeles complete the top five. These airports allow reaching the United States from South America and Asia, respectively. The top five European airports, Frankfurt, Charles de Gaulle, Ataturk, Heathrow, Amsterdam, are the primary hubs of the leading airlines in Europe, Lufthansa, Air France, Turkish Airlines, British Airways, and KLM, respectively. Note that Frankfurt and Amsterdam Schiphol airports are also among the five most important regional hubs. Charles de Gaulle Airport, the first French airport, is located in Paris, the world-leading destination in the world. Thanks to its central position, the Ataturk Airport is a vital hub where several air flights transit. Indeed, it is at a gateway for Asia, the Middle East, and Africa. The fourth inter-regional hub is London Heathrow, located in the top tourist city of the United Kingdom, London. It is the leading international airport in London.

While China dominates the regional hubs, inter-regional hubs are scattered in different countries of East and Southeast Asia. Beijing Capital Airport is the inter-regional hub. The Suvarnabhumi airport located in Bangkok, the capital city of Thailand, ranks second. It is an important hub serving local (Thai Airways and Bangkok Airways) and foreign airlines connecting to Asia, Oceania, Europe, and Africa. Narita, located in Tokyo, the capital of Japan, is a hub for Japan Air Lines and All Nippon Airways. The largest airport in South Korea, the Incheon Airport, is the fourth inter-regional hub. Located on the coast in Seoul, which receives a million tourist passengers per year, this airport is an important hub for Korean Air Lines. The Hong Kong Airport is the primary hub of Cathay Pacific and Dragon Air Lines, headquartered in Hong Kong.

The regional hubs, Dubai and Indira Gandhi Airport, are respectively the first and the fifth inter-regional hubs in the Africa-Middle-East Southern Asia area. The Hamad airport is the second inter-regional hub in this zone. This airport, located in Doha, the capital of Qatar, is a gateway to Europe, Asia, Africa, and Oceania. It is the most important hub for Qatar Airways. Abu Dhabi, the second-largest airport in the United Arab Emirates, is also strategically located. It is the primary hub of Etihad Airways. The fourth hub located in Cairo is the vital hub of Egypt Air.

In Oceania, the four regional hubs (Sydney K Smith Airport, Melbourne Airport, Auckland Airport, and Brisbane Airport) are also the inter-regional hubs. In addition, Perth Airport is an inter-regional hub. This airport, located on the coast of the capital city of Western Australia, is an essential airport for Alliance Airlines. One can see that Australian airports are the most influential in this area.

The two largest inter-regional hubs in South America (Guarulhos in Brazil and El Dorado in Colombia) are also the two largest regional hubs. The third hub, Jorge Chávez Airport, is located in Lima, the capital of Peru. It is the hub of different airlines such as ATSA, LATAM Perú Airlines. The largest airport in Argentina, the Ministro Pistarini Airport, located in the capital city of Argentina, has a vital role in international flights. It is the hub of Aerolíneas Argentinas.

In the Russia-Central Asia-Transcaucasia area, the top four inter-regional hubs are in Russia. Note that the top three are also present in the list of the top five regional hubs. The Vnukovo Airport is the fourth inter-regional airport. Located in Moscow, it is a hub for the Azur Air Lines. It corroborates the extreme centralization of the Russian air transport network in Moscow. The Heydar Aliyev Airport, located near the coast in Baku, the capital city of Azerbaïdjan, is the fifth inter-regional hub of the area. This airport is essential for Azerbaijan Air Lines.

### Comparison of the regional and inter-regional hubs with the hubs of the world air transportation network

We now compare the degree centrality of the nodes in the world air transportation network with the various degree centrality measures of the components. Table 9 reports information about the top 36 airports worldwide according to their total degree (the sum of their internal and external degree). It also contains their total degree, worldwide rank, local rank, and eventually global rank. For comparative purposes, airports are classified in the seven regions identified by the local components. At first glance, one can see that the distribution among regions is not homogeneous. Indeed, Europe and the North and Central America-Caribbean areas dominate, with 29 out of 36 airports in the world’s top ranks.

Thirteen airports of the North and Central America-Caribbean component belong to the top 36 of the world air transportation network. The top five are the top five regional airports. The eight other airports include the first five inter-regional hubs. The other three airports (Charlotte Douglas, McCarran, Detroit M Wayne) are regional hubs ranked 6, 7, and 9 in the local component. In this part of the world, regional hubs are more influential than inter-regional hubs. Indeed, the first inter-regional hub ranks nine in the world network. It shows that the air traffic in the North and Central America-Caribbean zone focuses mainly on regional destinations. The rank of the regional airports in the world air transportation network can be misleading because they are not the most active in inter-regional traffic.

With 16 airports, European airports are the most numerous in the top 36 in the world network. They are also among the most influential. Indeed, the top four world airports are in Europe. The top of the ranking includes the top inter-regional and regional airports (Frankfurt, Charles de Gaulle, Ataturk, London Heathrow, Amsterdam, Munich, London Stansted, Barcelona). Five regional airports complete this list. Three are in the UK (London Gatwick, Dublin, Manchester), one is in Germany (Dusseldorf), and one is in Austria (Vienna). Note that Dusseldorf uses to be the second airport in Germany before Munich took the lead. Two airports located in Rome and Madrid are more focused on inter-regional traffic. These results suggest that European airports are more oriented towards inter-regional connections.

There are four airports from the East and Southeast Asia area in the top 36 hubs of the world air network. Two are in the top five inter-regional hubs (Beijing Capital, Incheon), and two are in the leading regional hubs (Shanghai Pudong, Guangzhou Baiyun). Remember that Beijing Capital airport, which ranks seven in the world air network, is also the region’s top regional and inter-regional airport. This repartition between regional and inter-regional hubs indicates a balanced regional and inter-regional traffic dominated by China.

Dubai is the only airport in the top 36 world air network hubs for the Africa-Middle East-Southern Asia area. Ranked 10 in the world network, it is an essential regional hub (1st) and inter-regional hub (4th).

There is no airport of Oceania in Table 9. The first airport from this region, the Sydney K Smith Airport, ranks 84 worldwide. It is also the case for South America. Indeed, Guarulhos-G A F M, the highest degree airport in Brazil, ranks 74 in the world air network.

Two airports from the Russia-Central Asia-Transcaucasia zone are in the first 36 hubs of the global air network. These airports in Moscow are the most important hubs in the region at both regional and inter-regional levels.

To summarize, Europe and the United States dominate the world transportation network in terms of centrality. The main drawback of using world air transportation to evaluate the airports’ influence is that it cannot distinguish regional from inter-regional importance. Consequently, large parts of the world are hidden due to striking regional discrepancies. The component structure allows taking into account these regional disparities. Indeed, airports at the bottom in the world network ranking, which are very influential in their region, emerge. It is the case for airports such as the London Stansted Airport, the Chengdu Shuangliu Airport, King Abdulaziz Airport, Sydney K Smith Airport, Guarulhos-G A F M Airport, and the Pulkovo Airport that are the most connected in their region. Similarly, it brings to the attention that airports, such as Suvarnabhumi, Hamad, Melbourne, El Dorado, and Heydar Aliyev, are crucial for inter-regional traffic.

**Table 9:** The top 36 hubs of the world air transportation network

Region	Airport	City	Country	Degree	WR	LR	GR
North and central America- Caribbean	Hartsfield J Atlanta	Atlanta	USA	212	5	1	43
	Chicago O’Hare	Chicago	USA	200	6	3	39
	Dallas Fort Worth	Dallas	USA	188	8	2	78
	Denver	Denver	USA	165	12	4	197
	George B Houston	Houston	USA	165	12	5	66
	**John F Kennedy**	**New York**	**USA**	**160**	**14**	**15**	**9**
	**Newark Liberty**	**Newark**	**USA**	**152**	**19**	**10**	**24**
	**Lester B. Pearson**	**Toronto**	**Canada**	**147**	**22**	**13**	**19**
	**Los Angeles**	**Los Angeles**	**USA**	**144**	**24**	**11**	**30**
	Charlotte Douglas	Charlotte	USA	141	26	6	137
	**Miami**	**Miami**	**USA**	**138**	**30**	**14**	**27**
	McCarran	Las Vegas	USA	133	33	7	162
	Detroit M Wayne	Detroit	USA	133	33	9	120
Europe	***Schiphol***	***Amsterdam***	***Netherlands***	***242***	***1***	***1***	***7***
	***Frankfurt***	***Frankfurt***	***Germany***	***241***	***2***	***5***	***1***
	***Charles. de Gaulle***	***Paris***	***France***	***233***	***3***	***6***	***2***
	***Ataturk***	***Istanbul***	***Turkey***	***220***	***4***	***10***	***3***
	Munich	Munich	Germany	186	9	2	23
	**Heathrow**	**London**	**UK**	**167**	**10**	**33**	**4**
	L da Vinci-F	Rome	Italy	158	15	16	14
	London Gatwick	London	UK	157	17	6	45
	Barcelona	Barcelona	Spain	156	18	4	63
	Brussels	Brussels	Belgium	150	20	11	36
	Adolfo Suárez	Madrid	Spain	150	20	17	17
	Stansted	London	UK	143	25	3	46
	Dublin	Dublin	Ireland	141	26	8	101
	Düsseldorf	Düsseldorf	Germany	141	26	9	59
	Manchester	Manchester	UK	141	26	13	46
	Vienna	Vienna	Austria	136	31	14	50
East and Southeast Asia	***Beijing Capital***	***Beijing***	***China***	***194***	***7***	***1***	***10***
	Shanghai Pudong	Shanghai	China	147	22	3	28
	Guangzhou Baiyun	Guangzhou	China	135	32	2	52
	**Incheon**	**Seoul**	**South Korea**	**133**	**33**	**4**	**15**
Africa- Middle East- Southern Asia	***Dubai***	***Dubai***	***U A E***	***167***	***10***	***1***	***4***
Russia- Central Asia- Transcaucasia	***Domodedovo***	***Moscow***	***Russia***	***158***	***15***	***1***	***8***
	***Sheremetyevo***	***Moscow***	***Russia***	***133***	***33***	***3***	***6***

WR is the worldwide rank in the world air transportation network. It is computed using the total degree (internal and external). LR is the local rank computed in the large local components. GR is the global rank in the global component. No indication is given when an airport does not belong to the global component. Top inter-regional airports are in bold. Top regional and inter-regional airports are in bold and italic. Others are top regional airports

## Core analysis

In this section, we investigate the core structure of the components, and we perform a comparative analysis with the core of the world transportation network. To this end, we compute the max k-core. The k-core of a network is a sub-network in which a node has at least k neighbors. One can extract the maximum k-core by removing nodes iteratively from the network. Indeed, the 1-core, where each node has at least one link, is the whole network. One forms the 2-core by removing all the nodes with one connection in the 1-core network. Then, one extract the 3-core by removing all the nodes with two links in the 2-core, and so on until one reaches the core number max k-core for which it is impossible to obtain the (k+1)-core.

### Exploring the max k-core in the large local components

**Fig. 15:**
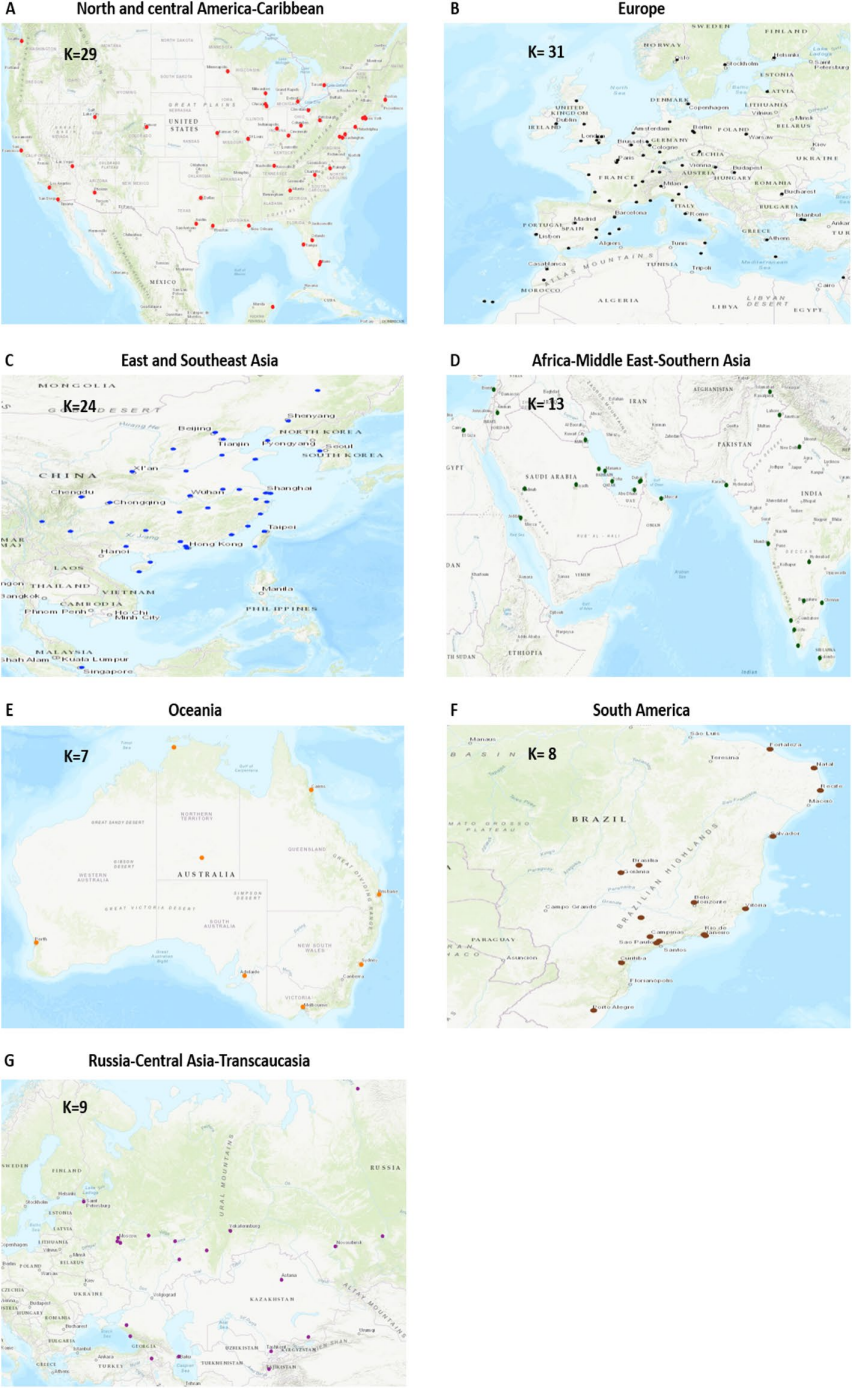
Core of the large local components. The dots represent the airports, and K is the maximum k-core. The core of the North and Central America-Caribbean component contains 42 airports. The core of Europe contains 65 airports. The core of East and Southeast Asia contains 38 airports. The core of Africa-Middle East-Southern Asia contains 26 airports. The core of Oceania contains 8 airports. The core of South America contains 16 airports. The core of Russia-Central Asia-Transcaucasia includes 19 airports

Figure 15 reports the airports included in the maximum k-core of the seven large local components with the value of the maximum k-core uncovered. In the following, we use indifferently the words max k-core and core for short.

The core of the North and Central America-Caribbean component contains 42 airports sharing at least 29 connections. Except for Lester B Pearson Airport (first local hub in Toronto, Canada) and the Cancun Airport (second hub in Mexico), these airports are in the USA. Table 10 (in the appendix) reports the list of airports included in the max k-core with their number of connections in the core, their rank in the local component, and their national rank according to their local degree centrality. One can see that top regional hubs outside the USA are not in the core. In addition, Nashville Airport, managed by Allegiant Air Lines, a low-cost airline, and Portland Airport, operated by Alaska Air Lines, is also absent from the core. In contrast, US domestic airports of minor importance in the local component but crucial at the national level integrate the core. They are mainly located on the East coast of the United States. The presence of the Lester B Pearson Airport and the Cancun Airport in the core reflects their high integration in the US transportation network. Indeed, Toronto is a main entry point to the US, and Cancun is a valued holiday destination in the US.

The core of the European component includes 65 airports. This result is close to the result reported in Lordan and Sallan (2017). Each airport in this core has at least 31 connections. In contrast with the North and Central America-Caribbean component, these airports are scattered in 27 countries. The European component is not very large compared to the others. One can consider it as a single geographic and economic space facilitating the connection of the member countries. The proportion of airports by country is not homogeneous. Germany, France, United Kingdom, Spain, and Italy dominate the core. Indeed, those are the major countries in Europe. The list of airports in the core correlates well with the top regional hubs of the component (See Table 11 in the appendix). Indeed, 59 airports ranked in the top 65 regional hubs belong to the core. The six missing airports are in Belgium (Brussels South Charleroi Airport), Italy (Il Caravaggio Airport), United Kingdom (Birmingham Airport, Glasgow Airport), Portugal (Faro Airport), and Netherlands (Eindhoven Airport). Although well-connected, these airports operate in the short and medium-haul segment, and they do not serve all of Europe. Six airports in the core are not included in 65 first regional hubs: Ben Gurion Airport, the first airport in Israel, Naples Airport, and Catania-Fontanarossa Airport in Italy, the Bordeaux-Merignac Airport and the Toulouse-Blagnac Airport in France, and the Menara Airport in Marrakech, Morocco. Except for Israel with more links with Europe than with the Middle East for political issues, those are mainly attractive regional tourist destinations (See Table 11 in the appendix).

The core of the East and Southeast Asia component includes 38 airports. Each airport has at least 24 links with the others. One observes a very similar behavior to the one encountered in the North and Central America-Caribbean component. Indeed, China, with 33 airports, dominates the core (See Table 12 in the appendix). These airports are in provincial and municipal capitals. Quite naturally, its “satellites” in the region belong to the core. Indeed, four airports are located in Hong Kong (Hong Kong Airport), Singapore (Singapore Changi Airport), Taiwan (Kaohsiung Airport and Taiwan Taoyuan Airport). Incheon Airport (South Korea) completes the core. Indeed, there is a vast community of Chinese people in Korea and strong economic and political relations between the two countries. Comparison with the top 38 regional airports (see Table 12 in the appendix) shows that South-East Asia’s leading regional airports (Malaysia, Thailand, Philippines, Vietnam) do not share enough links to belong to the core. It is also the case for Japan’s main airports due to the political divide.

In the Africa-Middle East-Southern Asia component, the core contains 26 airports distributed across twelve countries (See Table 13 in the appendix). At least, each airport in the core has 13 connections. These airports are essentially in the Middle East and Southern Asia. Indeed, the Arabic peninsula includes ten airports; one is in Jordan and one in Lebanon. Eight are in India, three in Pakistan, and one in Sri Lanka. There is no African airport in the core, except for Cairo, predominantly linked to the Arab world. There is also no airport from Iran. It must be related to the geopolitical context. The core airports reveal the significant air traffic between the Middle East and Southern Asia. Comparison with the top regional 26 airports reported in Table 13 shows that 20 airports in the core belong to the 26 top regional hub set. Leading regional airports of Iran (Mehrabad Airport and Mashhad airport), Ethiopia (Addis Ababa Bole Airport), Kenya (Jomo Kenyatta Airport), and Nigeria (Murtala Muhammed Airport) have less than 12 connections with the core airports. Consequently, Africa is not part of the component core.

There are eight airports in the core of the Oceania component (see Table 14 in the appendix). Each of them has seven connections. Thus, the core is a complete graph (*K*7). Once again, a country dominates the core. Indeed, all airports are in Australia. Three top regional hubs located in New Zealand (Auckland Airport), Papua New Guinea (Port Moresby Jacksons Airport), and French Polynesia (Faa’a Airport) are not in the core airports.

The South America component behaves similarly. Its core includes 16 airports situated in Brazil (See Table 15 in the appendix). These airports are mainly located on the East coast. The less connected core member has eight links. Comparisons with the top regional hubs reported in Table 15 show that six regional hubs do not have enough links with Brazilian airports to belong to the core. These are the airports from Argentina (Jorge Newbery Airpark, and Ministro Pistarini Airport), Columbia (El Dorado Airport), Peru (Jorge Chávez Airport), Chile (Comodoro A M Benítez Airport) and Brazil (Tancredo Neves Airport).

The core of the Russia-Central Asia-Transcaucasia component is also dominated by a single country Russia (see Table 16 in the appendix). Indeed, 14 airports are in Russia, 2 in Kazakhstan, and one in the capital cities of Uzbekistan, Tajikistan, Armenia, and Azerbaijan. Each airport shares at least nine connections with the core. Those ex-USSR countries have strong political relations that translate into solid interconnections between their metropolises. Note that four of the 19 top regional hubs from Russia are not in the core airports (Yakutsk Airport, Roshchino Airport, Irkutsk Airport, and Khabarovsk-Novy Airport).

To summarize, results show that leading countries widely influence the core of the local components. Indeed, the USA, China, Australia, Russia, Brazil dominate their region. The core reveals meaningful information about the national air traffic with its foreign satellite destinations. The core of the European component is more homogeneously distributed between various countries, even though major countries dominate the core. Finally, it appears that in the core of the Africa-Middle-East Southern -Asia component, Cairo is the unique core airport in Africa. The air integration concerns the Middle-east and Southern Asia essentially.

### Exploring the max k-core in the large global components

**Fig. 16:**
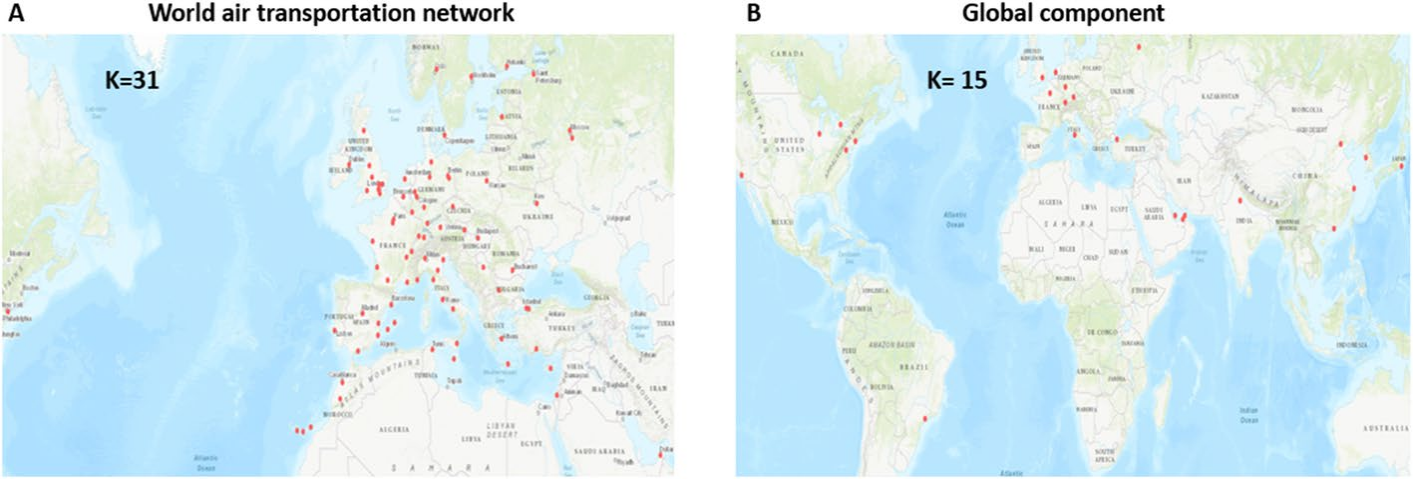
Figure **A** presents the 79 airports in the maximum k-core of the world air transportation network. Figure **B** presents the 24 airports in the maximum k-core of the large global component. The red dots are the airports and K is the maximum k-core

**Fig. 17:**
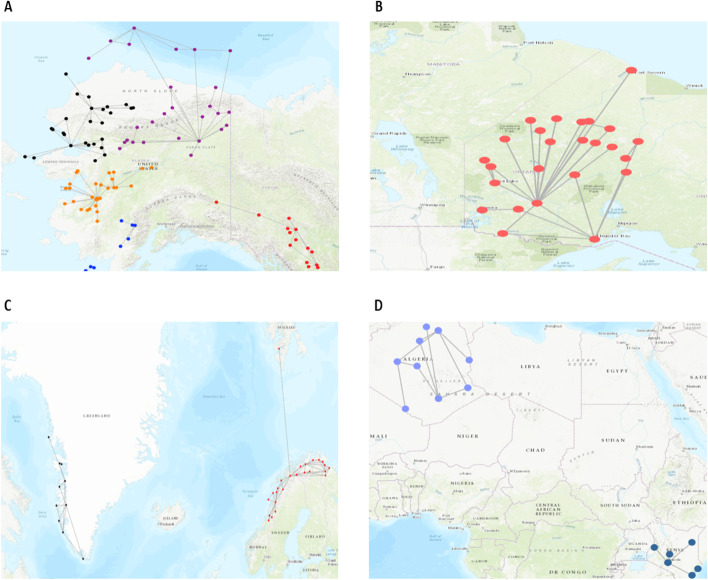
Small local components (size greater than 5 nodes). **A** Small components located in Alaska (USA). **B** small components in Ontario (Canada). **C** Small components in Green-Land and Norway (Europe). **D** Small component in Algeria and Kenya (Africa)

Figure 16b shows the core airports of the large global component. One can see that they cover the different regions of the world, except Oceania and Africa. Five global core airports are in North and Central America-Caribbean, nine in Europe, five in East and Southeast Asia, four in Africa-Middle East-Southern Asia, one in South America, and one in Russia-Central Asia-Transcaucasia. The maximum core of the global component contains 24 airports in 18 countries. These airports are mainly located in the capital of the countries and are managed by the largest airlines (see Table 17 in the appendix). Six of the top 24 inter-regional hubs are not at the core of the global component. These airports are in the United States (Newark Liberty), Spain (Adolfo S M–Barajas Airport), Thailand (Suvarnabhumi Airport), Singapore (Singapore Changi Airport), Malaysia (Kuala Lumpur Airport), and Russia (Pulkovo Airport). Although they have many links in the global component, most of these connections are not with major core airports. In contrast, some airports have fewer connections, but their links are with the core airports. It is the case for two airports in the North America-Caribbean region (Chicago O’Hare Airport, Los Angeles Airport, and Washington Dulles Airport), Switzerland (Zurich Airport), one in India (Indira Gandhi Airport), and one in Brazil (Guarulhos-Governador A F Montoro Airport). The less connected airports in the core have at least 15 long destinations throughout the world. It compares favorably with the core of a number of local components (Africa-Middle East-Southern Asia, Oceania, South America, Russia-Central Asia-Transcaucasia). Overall, Europe, North and Central America Caribbean, and East and Southeast Asia are particularly well connected.

### Comparison of max k-core of the components with the max k-core of the world air transportation network

This section compares the core of the world air transportation network with the core of the components. Figure 16a shows the core airports of the world air transportation network. It contains 79 airports listed in Table 18 in the appendix. Similar to the core of the European component, each airport has at least 31 links. Only five airports are not in the European local component. One is in the USA (John F Kennedy), one is in the Middle East (Dubai), and three are in Russia (Sheremetyevo, Pulkovo, and Domodedovo). Note that these top inter-regional hubs are in the gateway cities of their region. It also includes all the airports of the European local components. In addition, European capitals (Kyiv, Sofia, Belgrade), tourist destinations (Antalya, Larnaca, Tunis, Fuerteventura, Catania), and Hannover airport in Germany join the core. Note that Hannover is the base of low-cost companies serving metropolitan and leisure destinations.

Once again, these results reveal the importance of the component structure decomposition. Indeed, the core of the world transportation network brings to fore Europe, and it blurs the rest of the world. In contrast, the component structure allows dealing with the non-homogeneous structure of the world transportation networks, highlighting the similarities and the differences between the components.

## Conclusion

In this work, we present a mesoscale structure called the component structure. Each dense area of the network forms a local component. The subnetworks of nodes and links joining the local components are the global components. Although quite simple, this alternative mesoscopic representation presents multiple advantages. The exploration of the world air transportation network illustrates its usefulness. First of all, it allows partitioning the world air transportation network into meaningful regional entities. Indeed, similarly with communities, the local components do not partition the world according to strict geographical considerations but on the internal exchanges in a region.

Experiments reveal seven large local components corresponding to geographical area (North and Central America-Caribbean, Europe, East-Southeast Asia, Africa-Middle East-Southern Asia, Oceania, South America, and Russia-Central Asia-Transcaucasia) with blurred boundaries. Indeed, for example, some airports in North Africa belong to the European component, because they share more links with Europe than with Africa. Twenty small local components generally cover remote parts of a single country. There is one large global component distributed over the world. Eight small global components join a large local component with neighboring small local components or close airports at the periphery of their local components. The analysis of the component structure reveals the regional diversity of the world air transportation network. Nevertheless, the large components share some common characteristics. They are disassortative, small world, and have a heavy-tail degree and degree-degree distance distributions. A star-based topology generally characterizes the small components.

The comparative analysis of the degree centrality in the various components allows disentangling the diverse role of airports. Local component centrality measure highlights the regional importance of the airports, while the global component allows quantifying its influence in inter-regional exchanges. Note that some major airports are pretty active at both levels.

In the same vein, the core analysis reveals that considering the air transportation network as a whole can lead to misleading interpretation. Indeed, as Europe is more densely connected than the rest of the world, most airports in the core are in Europe. Failing to take into account the regional variations blur the picture. In contrast, core analysis of the components proves very instructive about various regional and inter-regional situations.

In future work, we plan to explore multiple directions. The most straightforward is to consider weighted and directed networks and other applications such as co-authorship networks analysis. One can also look at the hierarchical structure of the representation. Indeed, components are networks that can also be split into components and so on. It can be an excellent way to gain information about the fractal nature of real-world networks. The interplay between the core-periphery and the component structure is another direction of research.

## Data Availability

The datasets generated, used, and analyzed in the study are available from the corresponding author on reasonable request.

## Appendix

See Fig. 17, Table 19.

**Table 10:** Core airports of the North and Central America-Caribbean component (42)

Airport	City	Country	Core degree	Local rank	National rank
Hartsfield J Atlanta	Atlanta	United States	41	1	1
Dallas Fort Worth	Dallas-Fort Worth	United States	40	2	2
Chicago O’Hare	Chicago	United States	40	3	3
Denver	Denver	United States	41	4	4
George Bush Houston	Houston	United States	39	5	5
Charlotte Douglas	Charlotte	United States	41	6	6
McCarran	Las Vegas	United States	39	7	7
Minneapolis-St P/W-Chamberlain	Minneapolis	United States	41	8	8
Detroit M W County	Detroit	United States	41	9	9
Newark Liberty	Newark	United States	39	10	10
Los Angeles	Los Angeles	United States	40	11	11
Philadelphia	Philadelphia	United States	41	12	12
Lester B. Pearson	Toronto	Canada	38	13	13
Miami	Miami	United States	35	14	14
John F Kennedy	New York	United States	35	15	15
Orlando	Orlando	United States	40	16	16
Washington Dulles	Washington	United States	37	17	17
Salt Lake City	Salt Lake City	United States	31	18	18
Ronald Reagan Washington	Washington	United States	37	19	19
Phoenix Sky Harbor	Phoenix	United States	39	20	20
Fort Lauderdale Hollywood	Fort Lauderdale	United States	38	21	21
General E L Logan	Boston	United States	41	22	22
San Francisco	San Francisco	United States	37	23	23
Seattle Tacoma	Seattle	United States	31	25	24
Chicago Midway	Chicago	United States	31	26	25
La Guardia	New York	United States	29	27	26
Baltimore/W T Marshall	Baltimore	United States	38	28	27
Cancun	Cancun	Mexico	32	29	2
Tampa	Tampa	United States	36	30	28
St Louis Lambert	St. Louis	United States	39	32	29
San Diego	San Diego	United States	30	35	31
Cleveland Hopkins	Cleveland	United States	35	37	32
Nashville	Nashville	United States	36	38	34
Cincinnati N Kentucky	Cincinnati	United States	33	40	35
Kansas City	Kansas City	United States	35	42	36
**Austin Bergstrom**	**Austin**	**United States**	**30**	**45**	**38**
**Louis A N Orleans**	**New Orleans**	**United States**	**35**	**47**	**39**
**Raleigh-durham**	**Raleigh-durham**	**United States**	**34**	**48**	**40**
**Pittsburgh**	**Pittsburgh**	**United States**	**31**	**50**	**41**
**Indianapolis**	**Indianapolis**	**United States**	**30**	**53**	**44**
**John Glenn Columbus**	**Columbus**	**United States**	**29**	**55**	**45**
**General Mitchell**	**Milwaukee**	**United States**	**29**	**64**	**50**
***Licenciado Benito Juarez***	***Mexico City***	***Mexico***	-	***24***	***1***
***Montreal/Pierre Elliott Trudeau***	***Montreal***	***Canada***	-	***31***	***2***
***Vancouver***	***Vancouver***	***Canada***	–	***33***	***3***
***Portland***	***Portland***	***United States***	–	***34***	***30***
***Calgary***	***Calgary***	***Canada***	–	***36***	***4***
***William P Hobby***	***Houston***	***United States***	–	***38***	***33***
***Tocumen***	***Panama City***	***Panama***	–	***41***	***1***

Airports not in the top 42 regional hubs are in bold. The core degree of a node is its number of links in the maximum k-core. Local rank is the rank in the large local component. National rank is the airport rank in its country. Airports in the top 42 regional hubs and not in the k-core are in bolditalic

**Table 11:** Core airports of the European component (65)

Airport	City	Country	Core degree	Local rank	National rank
Schiphol	Amsterdam	Netherlands	62	1	1
Munich Airport	Munich	Germany	61	2	1
London Stansted	London	UK	34	3	1
Barcelona	Barcelona	Spain	62	4	1
Frankfurt	Frankfurt	Germany	54	5	2
London Gatwick	London	UK	52	6	2
Charles de Gaulle	Paris	France	56	7	1
Dublin	Dublin	Ireland	58	8	1
Duesseldorf	Duesseldorf	Germany	48	9	3
Ataturk	Istanbul	Turkey	48	10	1
Brussels	Brussels	Belgium	57	11	1
Palma De Mallorca	Palma de Mallorca	Spain	42	12	2
Manchester	Manchester	UK	49	13	3
Vienna	Vienna	Austria	49	14	1
Malaga	Malaga	Spain	49	15	3
Leonardo da V–F	Rome	Italy	57	16	1
Madrid–Barajas	Madrid	Spain	58	17	4
Kastrup	Copenhagen	Denmark	52	18	1
S Arlanda	Stockholm	Sweden	46	19	1
Paris-Orly	Paris	France	39	20	2
Zurich	Zurich	Switzerland	54	21	1
Berlin-Tegel	Berlin	Germany	47	22	4
Alicante	Alicante	Spain	32	23	5
E. Venizelos	Athens	Greece	44	24	1
Oslo Lufthavn	Oslo	Norway	42	25	1
Geneva Cointrin	Geneva	Switzerland	53	26	2
Cologne Bonn	Cologne	Germany	37	27	5
London Luton	London	UK	34	28	4
Edinburgh	Edinburgh	UK	43	29	5
Nice-Côte d’Azur	Nice	France	46	30	3
Hamburg	Hamburg	Germany	47	31	6
Stuttgart	Stuttgart	Germany	33	32	7
London Heathrow	London	UK	47	33	6
H. Delgado	Lisbon	Portugal	51	34	1
Tenerife South	Tenerife	Spain	38	36	6
Vaclav Havel	Prague	Czech Republic	52	37	1
Marseille	Marseille	France	36	38	4
Gran Canaria	Gran Canaria	Spain	38	39	7
Malpensa	Milano	Italy	51	40	2
Saint-Exupéry	Lyon	France	37	42	5
Sabiha Gökçen	Istanbul	Turkey	31	43	2
Bristol	Bristol	UK	34	44	7
Warsaw Chopin	Warsaw	Poland	33	46	1
Liszt Ferenc	Budapest	Hungary	46	47	1
Basel-Mulhouse	Mulhouse	France	40	48	6
Malta	Malta	Malta	39	49	1
Venice Marco Polo	Venice	Italy	42	51	5
Berlin-Schonefeld	Berlin	Germany	35	52	8
G Marconi	Bologna	Italy	36	53	6
Pisa	Pisa	Italy	31	54	7
Ibiza	Ibiza	Spain	31	55	8
Helsinki Vantaa	Helsinki	Finland	35	56	1
Henri Coandaf	Bucharest	Romania	34	57	1
Riga	Riga	Latvia	34	58	1
Valencia	Valencia	Spain	31	59	9
N Kazantzakis	Heraklion	Greece	31	60	2
Nantes Atlantique	Nantes	France	34	62	7
Mohammed V	Casablanca	Morocco	31	63	1
Luxembourg-Findel	Luxemburg	Luxemburg	31	65	1
**Ben Gurion**	**Tel-aviv**	**Israel**	**38**	**70**	**1**
**Naples**	**Naples**	**Italy**	**35**	**72**	**6**
**Bordeaux-Merignac**	**Bordeaux**	**France**	**31**	**76**	**8**
**Catania-Fontanarossa**	**Catania**	**Italy**	**35**	**77**	**7**
**Menara**	**Marrakech**	**Morocco**	**32**	**80**	**2**
**Toulouse-Blagnac**	**Toulouse**	**France**	**31**	**82**	**10**
***Brussels South Charleroi***	***Charleroi***	***Brussels***	–	***35***	***2***
***Il Caravaggio***	***Bergamo***	***Italy***	–	***41***	***3***
***Birmingham***	***Birmingham***	***UK***	–	***45***	***8***
***Faro***	***Faro***	***Portugal***	–	***50***	***2***
***Glasgow***	***Glasgow***	***UK***	–	***61***	***9***
***Eindhoven***	***Eindhoven***	***Netherlands***	–	***64***	***2***

Airports not in the top 65 regional hubs are in bold. The core degree of a node is its number of links in the maximum k-core. Local rank is the rank in the large local component. National rank is the airport rank in its country. Airports in the top 65 regional hubs and not in the k-core are in bolditalic

**Table 12:** Core airports of the East and Southeast Asia component (38)

Airport	City	Country	Core degree	Local rank	National rank
Beijing Capital Airport	Beijing	China	36	1	1
Guangzhou Baiyun	Guangzhou	China	35	2	2
Shanghai Pudong	Shanghai	China	33	3	3
Chengdu Shuangliu	Chengdu	China	36	4	4
Taiwan Taoyuan	Taipei	Taiwan	35	5	1
Kunming Changshui	Kunming	China	35	6	5
Incheon	Seoul	South Korea	29	7	1
Hong Kong	Hong Kong	Hong Kong	36	8	1
Shenzhen Bao’an	Shenzhen	China	34	9	6
Chongqing Jiangbei	Chongqing	China	36	10	7
Xi’an Xianyang	Xi’an	China	33	11	8
Singapore Changi	Singapore	Singapore	24	12	1
Hangzhou Xiaoshan	Hangzhou	China	29	13	12
Xiamen Gaoqi	Xiamen	China	33	14	13
Changsha Huanghua	Changcha	China	32	17	14
Shanghai Hongqiao	Shanghai	China	26	19	15
Wuhan Tianhe	Wuhan	China	32	21	16
Taoxian	Shenyang	China	26	22	17
Zhoushuizi	Dalian	China	25	23	18
Liuting	Qingdao	China	31	24	19
Nanjing Lukou	Nanjing	China	29	25	20
Zhengzhou Xinzheng	Zhengzhou	China	30	26	21
Taiping	Harbin	China	24	27	22
Tianjin Binhai	Tianjin	China	29	29	23
Fuzhou Changle	Fuzhou	China	29	32	24
Longdongbao	Guiyang	China	29	34	25
Taiyuan Wusu	Taiyuan	China	28	35	26
Sanya Phoenix	Sanya	China	28	37	27
Nanning Wuxu	Nanning	China	28	38	28
**Ningbo Lishe**	**Ninbo**	**China**	**26**	**41**	**29**
**Nanchang Changbei**	**Nanchang**	**China**	**29**	**44**	**30**
**Kaohsiung**	**Kaohsiung**	**Taiwan**	**24**	**45**	**31**
**Haikou Meilan**	**Haikou**	**China**	**28**	**46**	**32**
**Yaoqiang**	**Jinan**	**China**	**27**	**50**	**33**
**Hefei Luogang**	**Hefei**	**China**	**26**	**52**	**34**
**Wenzhou Longwan**	**Wenzhou**	**China**	**24**	**53**	**35**
**Guilin Liangjiang**	**Guilin**	**China**	**26**	**55**	**36**
**Lijiang**	**Lijiang**	**China**	**24**	**61**	**37**
***Suvarnabhumi***	***Kuala Lumpur***	***Malaysia***	–	***15***	***1***
***Suvarnabhumi***	***Bangkok***	***Thailand***	–	***16***	***2***
***Tokyo Haneda***	***Tokyo***	***Japan***	–	***18***	***1***
***Ninoy Aquino***	***Manila***	***Philippines***	–	***20***	***1***
***Don Mueang***	***Bangkok***	***Thailand***	–	***28***	***2***
***Narita***	***Tokyo***	***Japan***	–	***28***	***2***
***Kansai***	***Osaka***	***Japan***	–	***31***	***3***
***Tan Son Nhat***	***Ho Chi Minh City***	***Vietnam***	–	***33***	***1***
***Noi Bai***	***Hanoi***	***Vietnam***	–	***36***	***2***

Airports not in the top 38 regional hubs are in bold. The core degree of a node is its number of links in the maximum k-core. Local rank is the rank in the large local component. National rank is the airport rank in its country. Airports in the top 38 regional hubs and not in the k-core are in bolditalic

**Table 13:** Core airports of the Africa-Middle East-Southern Asia component (26)

Airport	City	Country	Core degree	Local rank	National rank
Dubai	Dubai	United Arab Emirates	22	1	1
King Abdulaziz	Jeddah	Saudi Arabia	23	2	2
Indira Gandhi	Delhi	India	19	3	1
Chhatrapati Shivaji	Mumbai	India	20	4	2
Sharjah	Sharjah	United Arab Emirates	23	6	2
King Khaled	Riyadh	Saudi Arabia	24	7	2
Hamad	Doha	Qatar	19	9	1
Muscat	Muscat	Oman	25	10	1
Kuwait	Kuwait	Kuwait	22	11	1
King Fahd	Dammam	Saudi Arabia	24	12	3
Abu Dhabi	Abu Dhabi	United Arab Emirates	23	13	3
Cairo	Cairo	Egypt	14	14	1
Bahrain	Bahrain	Bahrain	22	15	1
Kempegowda	Bangalore	India	16	17	3
Queen Alia	Amman	Jordan	15	20	1
Chennai	Madras	India	18	21	4
Jinnah	Karachi	Pakistan	14	22	1
Prince Mohammad Bin Abdulaziz	Madinah	Saudi Arabia	16	24	3
Beirut Rafic Hariri	Beirut	Lebanon	13	25	1
Rajiv Gandhi	Hyderabad	India	13	26	6
**Bandaranaike Colombo**	**Colombo**	**Sri Lanka**	**17**	**27**	**1**
**Benazir Bhutto**	**Islamabad**	**Pakistan**	**13**	**28**	**2**
**Alama Iqbal**	**Lahore**	**Pakistan**	**14**	**33**	**3**
**Cochin**	**Kochi**	**India**	**17**	**34**	**7**
**Calicut**	**Calicut**	**India**	**14**	**37**	**8**
**Trivandrum**	**Trivandrum**	**India**	**14**	**38**	**9**
***Addis Ababa Bole***	***Addis Ababa***	***Ethiopia***	–	***5***	***1***
***Jomo Kenyatta***	***Nairobi***	***Kenya***	–	***8***	***2***
***Mehrabad***	***Teheran***	***Iran***	–	***16***	***1***
***Mashhad***	***Mashhad***	***Iran***	–	***18***	***2***
***Murtala Muhammed***	***Lagos***	***Nigeria***	–	***19***	***1***
***Netaji Subhash Chandra Bose***	***Kolkata***	***India***	–	***23***	***5***

Airports not in the top 26 regional hubs are in bold. The core degree of a node is its number of links in the maximum k-core. Local rank is the rank in the large local component. National rank is the airport rank in its country. Airports in the top 26 regional hubs and not in the k-core are in bolditalic

**Table 14:** Core airports of the Oceania component (8)

Airport	City	Country	Core degree	Local rank	National rank
Sydney Kingsford Smith	Sydney	Australia	7	1	1
Brisbane	Brisbane	Australia	7	2	2
Melbourne	Melbourne	Australia	7	4	3
Cairns	Cairns	Australia	7	6	4
Perth	Perth	Australia	7	7	5
**Adelaide**	**Adelaide**	**Australia**	**7**	**10**	**6**
**Darwin**	**Darwin**	**Australia**	**7**	**15**	**7**
**Alice Springs**	**Alice Springs**	**Australia**	**7**	**18**	**10**
***Auckland***	***Auckland***	***New Zealand***	–	***3***	***1***
***Port Moresby Jacksons***	***Port Moresby***	***Papua New Guinea***	–	***5***	***1***
***Faa’a***	***Papeete***	***French Polynesia***	–	***8***	***1***

Airports not in the top 8 regional hubs are in bold. The core degree of a node is its number of links in the maximum k-core. Local rank is the rank in the large local component. National rank is the airport rank in its country. Airports in the top 8 regional hubs and not in the k-core are in bolditalic

**Table 15:** Core airports of the South America component (16)

Airport	City	Country	Core degree	Local rank	National rank
Guarulhos-Governador André Franco Montoro	Sao Paulo	Brazil	13	1	1
Viracopos	Campinas	Brazil	13	3	2
Presidente Juscelino Kubistschek	Brasilia	Brazil	14	5	3
Rio Galeao–Tom Jobim	Rio De Janeiro	Brazil	14	6	4
Tancredo Neves	Belo Horizonte	Brazil	15	7	5
Congonhas	Sao Paulo	Brazil	10	10	6
Deputado Luiz E Magalhaes	Salvador	Brazil	12	11	7
Salgado Filho	Porto Alegre	Brazil	8	13	8
Afonso Pena	Curitiba	Brazil	10	14	9
Santos Dumont	Rio De Janeiro	Brazil	11	15	10
**Pinto Martins**	**Fortaleza**	**Brazil**	**8**	**17**	**12**
**Guararapes-Gilberto Freyre**	**Recife**	**Brazil**	**8**	**18**	**13**
**Santa Genoveva**	**Goiania**	**Brazil**	**8**	**25**	**16**
**Eurico de Aguiar Salles**	** Vitoria**	**Brazil**	**8**	**26**	**17**
**Governador Aluízio Alves**	**Natal**	**Brazil**	**8**	**29**	**18**
**Leite Lopes**	**Ribeirao Preto**	**Brazil**	**8**	**32**	**20**
***El Dorado***	***Bogota***	***Colombia***	–	***2***	***1***
***Jorge Newbery Airpack***	***Buenos Aires***	***Argentina***	–	***4***	***1***
***Jorge Chavez***	***Santiago***	***Chile***	–	***9***	***1***
***Ministro Pistarini***	***Buenos Aires***	***Argentina***	–	***12***	***2***
***Tancredo Neves***	***Belo Horizon***	***Brazil***	–	***7***	***5***

Airports not in the top 16 regional hubs are in bold. The core degree of a node is its number of links in the maximum k-core. Local rank is the rank in the large local component. National rank is the airport rank in its country. Airports in the top 16 regional hubs and not in the k-core are in bolditalic

**Table 16:** Core airports of the Russia-Central Asia- Transcaucasia component (19)

Airport	City	Country	Core degree	Local rank	National rank
Domodedovo	Moscow	Russia	17	1	1
Pulkovo	St. Petersburg	Russia	18	2	2
Sheremetyevo	Moscow	Russia	15	3	3
Tolmachevo	Novosibirsk	Russia	14	4	4
Koltsovo	Yekaterinburg	Russia	18	5	5
Tashkent	ashkent	Uzbekistan	16	6	1
Vnukovo	Moscow	Russia	12	7	6
Astana	Tselinograd	Kazakhstan	9	8	1
Almaty	Alma-ata	Kazakhstan	10	9	2
Yemelyanovo	Krasnoyarsk	Russia	13	10	7
Zvartnots	Yerevan	Armenia	11	13	1
Krasnodar Pashkovsky	Krasnodar	Russia	11	11	8
Dushanbe	Dushanbe	Tajikistan	12	15	1
Ufa	Ufa	Russia	9	16	9
Heydar Aliyev	Baku	Azerbaijan	10	18	1
Sochi	Sochi	Russia	12	19	9
**Kazan**	**Kazan**	**Russia**	**9**	**22**	**11**
**Nizhny Novgorod Strigino**	**Nizhniy Novgorod**	**Russia**	**9**	**23**	**12**
**Kurumoch**	**Samara**	**Russia**	**10**	**24**	**13**
**Norilsk-Alykel**	**Norilsk**	**Russia**	**9**	**26**	**14**
***Yakutsk***	***Yakutsk***	***Russia***	–	***12***	***9***
***Roshchino***	***Tyumen***	***Russia***	–	***14***	***10***
***Irkutsk***	***Irkutsk***	***Russia***	–	***17***	***13***
***Khabarovsk-Novy***	***Khabarovsk***	***Russia***	–	***20***	***18***

Airports not in the top 16 regional hubs are in bold. The core degree of a node is its number of links in the maximum k-core. Local rank is the rank in the large local component. National rank is the airport rank in its country. Airports in the top 19 regional hubs and not in the k-core are in bolditalic

**Table 17:** Core airports of the global component (24)

Airport	City	Country	Core degree	Global rank
Frankfurt am Main	Frankfurt	Germany	16	1
Charles de Gaulle	Paris	France	16	2
Atatürk	Istanbul	Turkey	16	3
London Heathrow	London	United Kingdom	16	4
Dubai	Dubai	United Arab Emirates	19	5
Sheremetyevo	Moscow	Russia	19	6
Amsterdam Schiphol	Amsterdam	Netherlands	16	7
John F Kennedy	New York	United States	19	9
Beijing Capital	Beijing	China	18	10
Hamad	Doha	Qatar	17	11
Narita	Tokyo	Japan	17	13
Leonardo da Vinci–Fiumicino	Rome	Italy	16	14
Incheon	Seoul	South Korea	18	15
Hong Kong	Hong Kong	Hong Kong	17	16
Lester B. Pearson	Toronto	Canada	17	19
Abu Dhabi	Abu Dhabi	United Arab Emirates	17	22
Munich	Munich	Germany	15	23
**Guarulhos-Governador A F Montoro**	**Sao Paulo**	**Brazil**	**16**	**26**
**Shanghai Pudong**	**Shanghai**	**China**	**17**	**28**
**Zurich**	**Zurich**	**Switzerland**	**15**	**29**
**Los Angeles**	**Los Angeles**	**United States**	**16**	**30**
**Indira Gandhi**	**Delhi**	**India**	**16**	**32**
**Washington Dulles**	**Washington**	**United States**	**16**	**38**
**Chicago O’Hare**	**Chicago**	**United States**	**17**	**39**
***Domodedovo***	***Moscow***	***Russia***	–	***8***
***Suvarnabhumi***	***Bangkok***	***Thailand***	–	***12***
***Adolfo S Madrid–Barajas***	***Madrid***	***Spain***	–	***17***
***Pulkovo***	***St. Petersbourg***	***Russia***	–	***18***
***Singapore Changi***	***Singapoer***	***Singapore***	–	***20***
***Kuala Lumpur***	***Kuala Lumpur***	***Malaysia***	–	***21***
***Newark Liberty***	***Newark***	***United States***	–	***24***

Airports not in the top 24 inter-regional hubs are in bold. The core degree of a node is its number of links in the maximum k-core. Global rank is the rank in the large local component. Airports in the top 24 inter-regional hubs and not in the k-core are in bolditalic

**Table 18:** These airports are not in the core of the European local component, but they belong to the core of the world air transportation network

Airport	City	Country	Core degree	Worldwide rank	Local rank	Global rank
***Dubai***	***Dubai***	***United Arab Emirates***	***40***	***10***	***1***	***5***
***John F Kennedy***	***New York***	***United States***	***32***	***14***	***15***	***9***
***Domodedovo***	***Moscow***	***Russia***	***35***	***16***	***1***	***8***
***Pulkovo***	***St. Petersburg***	***Russia***	***36***	***63***	***2***	***18***
***Sheremetyevo***	***Moscow***	***Russia***	***49***	***36***	***3***	***6***
Boryspil	Kiev	Ukraine	35	145	86	91
Tunis Carthage	Tunis	Tunisia	32	165	103	86
Sofia	Sofia	Bulgaria	31	218	96	305
Fuerteventura	Fuerteventura	Spain	31	158	65	-
Antalya	Antalya	Turkey	31	137	65	156
Larnaca	Larnaca	Cyprus	33	156	88	111
Belgrade Nikola Tesla	Belgrade	Serbia	31	178	81	238
Hannover	Hannover	Germany	32	171	75	476
Catania-Fontanarossa	Catania	Italy	37	177	75	440

Airports in the European local component are in black. Airports from other local components are in bolditalic. The core degree is the number of links in the maximum k-core network. Worldwide rank is the rank in the world air transportation network. Local rank is the rank in the large local components. Global rank is the rank in the global component

**Table 19:** Small global components

Airport	City	Country	Local component number
Postville	Postville	Canada	10 (Canada)
Goose Bay	Goose Bay	Canada	North and Central America-Caribbean
Val-d’Or	Val D’or	Canada	North and Central America-Caribbean
Chisasibi	Chisasibi	Canada	7 (Canada)
Waskaganish	Waskaganish	Canada	7 (Canada)
Natashquan	Natashquan	Canada	9 (Canada)
Sept-Iles	Sept-Iles	Canada	North and Central America-Caribbean
Lourdes de Blanc Sablon	Lourdes-De-Blanc-Sablon	Canada	North and Central America-Caribbean
St Augustin	St-Augustin	Canada	9 (Canada)
Fort Chipewyan	Fort Chipewyan	Canada	7 (Canada)
Fort McMurray	Fort Mcmurray	Canada	North and Central America-Caribbean
Timmins/Victor M. Power	Timmins	Canada	North and Central America-Caribbean
Moosonee	Moosonee	Canada	9 (Canada)
Nouadhibou	Nouadhibou	Mauritania	17 (Mauritania)
Gran Canaria	Gran Canaria	Spain	Europe
Dongshan	Hailar	China	East and Southeast Asia
Chita-Kadala	Chita	Russia	Russia-Central Asia-Transcaucasia

The Local component number corresponds to the number in Fig. 10

## Abbreviations

OAG: Official Aviation Guide
LPA: Label Propagation Algorithm
NMI: Normalized Mutual Information
TPL: Truncated Power Law
PL: Power Law
LN: Log Normal
S-E: Stretched Exponential
ID: Internal Degree
LR: Local Rank
CP: Cumulative fraction of connected airports
ED: External Degree
GRR: Global Rank of the airport in the Region
GRC: Global Rank in the global Component
WR: Worldwide Rank
GR: Global Rank
